# Host-encoded ETP2 is involved in recruiting the dynamin-like protein ETP9 to the endosymbiont division site in trypanosomatid *Angomonas deanei*

**DOI:** 10.1128/mbio.02247-25

**Published:** 2025-09-12

**Authors:** Anay K. Maurya, Lawrence Rudy Cadena, Georg Ehret, Eva C. M. Nowack

**Affiliations:** 1 Department of Biology, Institute of Microbial Cell Biology, Heinrich Heine University Düsseldorf 90266, Düsseldorf, Germany; Max Planck Institute for Chemical Ecology, Jena, Germany

**Keywords:** endosymbiosis, organellogenesis, evolution, cell cycle synchronization, FtsZ

## Abstract

A single β-proteobacterial endosymbiont, *Candidatus* Kinetoplastibacterium crithidii, resides in the cytosol of the trypanosomatid *Angomonas deanei* and divides at a defined stage of its host’s cell cycle. This endosymbiont has a highly reduced genome of 0.8 Mb and, notably, has lost most essential bacterial division genes, resulting in a loss of division autonomy. It has been previously demonstrated that a host-encoded dynamin-like protein, endosymbiont-targeted host protein (ETP)9, plays an indispensable role in the division of the endosymbiont. In this study, we identified a second nucleus-encoded component of the endosymbiont division machinery, termed ETP2, currently annotated as a “hypothetical protein.” We observed that ETP2 arrives before ETP9 at the bacterial division site. ETP2 deletion or depletion results in division phenotypes with long, filamentous endosymbionts accompanied by severely distorted host cells or host daughter cells lacking endosymbionts. We found that ETP2 depletion results in mis-localization of ETP9, whereas ETP2 localization to the endosymbiont division site is independent of ETP9. *In silico* analyses revealed that ETP2 is found exclusively in endosymbiont-harboring trypanosomatids of the subfamily Strigomonadinae and is most likely an intrinsically disordered protein. Collectively, our data suggests that ETP2 is an integral component of the endosymbiont division machinery involved in recruiting ETP9 to the division site. This finding highlights the evolution of a complex host-derived molecular mechanism that exerts tight control over its endosymbiont without requiring gene transfers from the bacterium.

**IMPORTANCE:**

The ancient uptake and transformation of free-living bacteria into eukaryotic organelles involved extensive structural, physiological, and genetic changes. More recently established endosymbioses offer a unique opportunity to observe intermediate stages in the complex process by which a prokaryote becomes genetically integrated into a eukaryotic cell. Hence, studying the molecular mechanisms that govern host-endosymbiont interactions holds the potential for uncovering the scenarios and molecular processes behind organelle formation. The trypanosomatid *Angomonas deanei* has been recently reported to manifest nuclear control over its endosymbiont’s division. In this study, we identified and characterized a new nucleus-encoded component of the endosymbiont division machinery. This study further supports that a novel intermediate between endosymbiont and organelle evolved in *A. deanei* and provides new leverage to entangle the evolution of its fascinating nucleus-controlled endosymbiont division machinery.

## INTRODUCTION

Mitochondria and plastids originated from bacteria. However, after over a billion years of co-evolution with their host, the organelles’ proteome composition, metabolic activity, and division are predominantly regulated by the genetic instructions of the nucleus. More recently established endosymbioses offer the opportunity to investigate intermediate stages in organellogenesis (1–3).

The trypanosomatid *Angomonas deanei* contains a single β-proteobacterial endosymbiont that supplies its host with diverse metabolites and co-factors (4–7). Intriguingly, this endosymbiont, *Candidatus* Kinetoplastibacterium crithidii, divides at a specific point in the host cell cycle (8). The presence of *Ca*. K. crithidii seems to be obligate in some *A. deanei* strains, including *A. deanei* American Type Culture Collection (ATCC) PRA-265 (9). However, in other strains, aposymbiotic cells can be generated through antibiotic treatment (6).

*Ca*. Kinetoplastibacterium was apparently acquired ~40 to 120 million years ago by a common ancestor of the genera *Angomonas*, *Strigomonas*, and *Kentomonas* that together form the subfamily Strigomonadinae (10–12). All *Ca*. Kinetoplastibacterium endosymbionts underwent pronounced reductive genome evolution (4, 13), resulting in the loss of most essential bacterial division genes (9, 14). However, the *ftsZ* gene has been retained. FtsZ is a polymer-forming GTPase that, in bacteria, plastids, and some mitochondria, forms the so-called Z-ring at the inner face of the inner membrane (15, 16). In bacteria, the Z-ring serves as a scaffold for the assembly of the machinery that synthesizes the division septum. In plastids, the Z-ring forms a complex with proteins in the inner and outer envelope membrane that confers topological information to the outer face of the outer envelope membrane and recruits a soluble dynamin-like protein (DLP) that forms a contractile ring structure around the plastid. Reciprocal interactions between the Z-ring and DLP ring result in a concerted constriction of the division machinery responsible for plastid fission (17).

Recently, we identified seven endosymbiont-targeted host proteins (ETPs) in *A. deanei* (18, 19). One of these proteins is the DLP ETP9 that apparently forms a contractile ring around the endosymbiont division site (ESDS) and takes part in endosymbiont division in a manner analogous to the division of mitochondria and plastids (9, 18). Attempts to generate homozygous ETP9 knockouts, in which both *etp9* alleles are deleted, proved unsuccessful, suggesting an essential function for ETP9. Knockdown (KD) experiments demonstrated that despite the formation of Z-ring structures in ETP9-depleted cells, endosymbionts are divisionimpaired, forming filamentous structures, within distorted host cells (9). How ETP9 is recruited to the ESDS, if it acts alone or is part of a more complex division machinery, and if nucleus-encoded endosymbiont division factors are physically linked to the bacterial Z-ring are currently unknown.

Notably, ETP9 is not the sole ETP that localizes at the ESDS. ETP2 (annotated as a “hypothetical protein”) and ETP7 (containing a predicted phage-tail lysozyme domain, GenBank accession number CAD2217314.1) exhibited similar localizations (18). ETP2 was originally identified as CAD2221027.1; however, later, the available gene model was corrected based on comparison to transcriptome data and genome assembly LXWQ00000000 (19) (see Fig. S1). Here, we investigated the role of ETP2.

## RESULTS

### Recombinant eGFP-ETP2 is functional and consistently localizes at the ESDS

**Fig 1:**
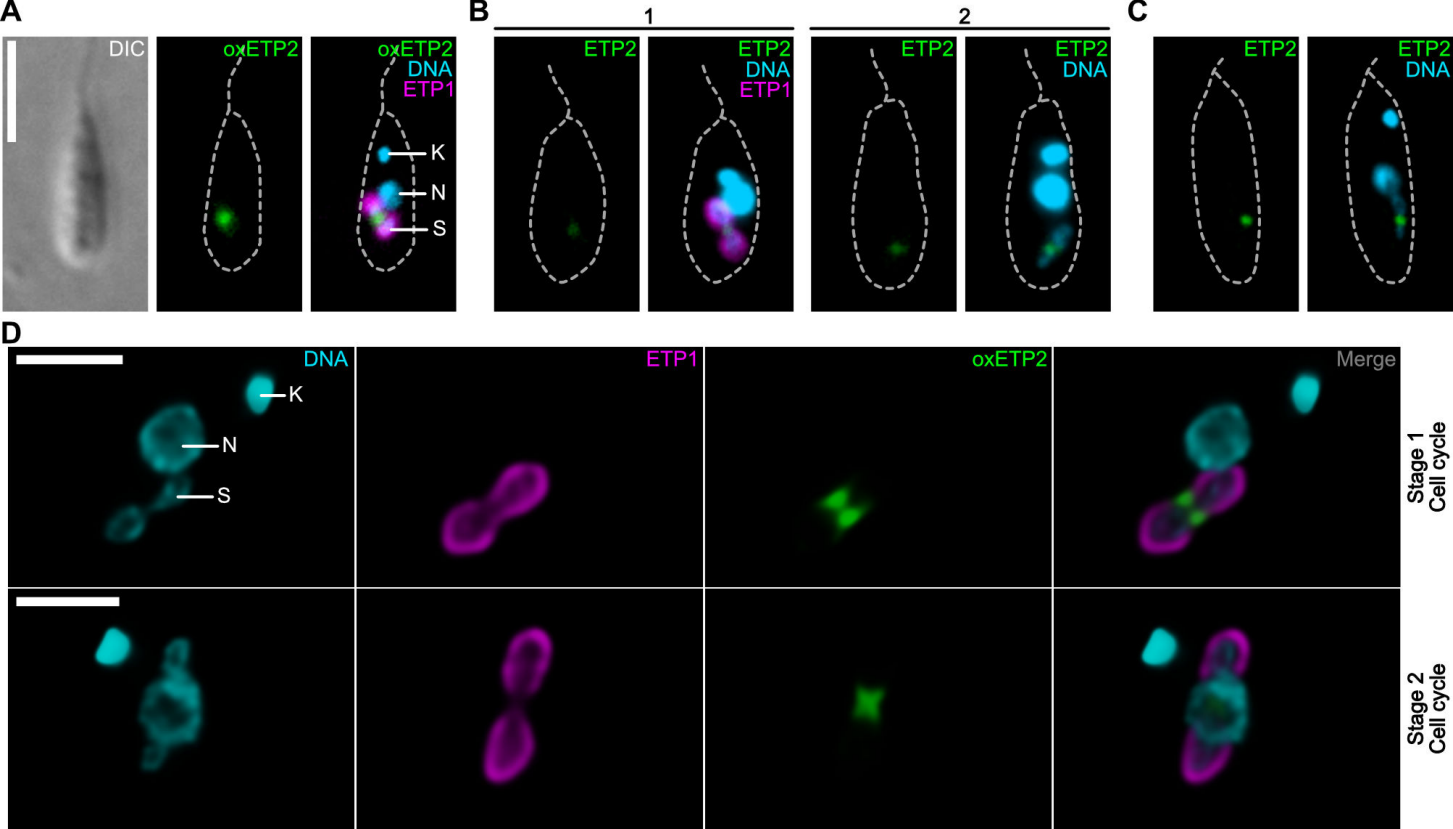
Subcellular localization of recombinant ETP2 in *A. deanei*. (**A–C**) Epifluorescence microscopic analysis of subcellular localization of (**A**) eGFP-ETP2 overexpressed (oxETP2) from the *δ-amastin* locus in a cell co-expressing the endosymbiont marker mScarlet-ETP1; (**B**) eGFP-ETP2 expressed from its endogenous locus in the background of a cell expressing mScarlet-ETP1 (micrograph 1) or the Wt background (micrograph 2); and (**C**) eGFP-ETP2 expressed from both endogenous loci. For each image set, the eGFP channel alone (left) and the overlay of channels (right) for Hoechst 33342-stained DNA, eGFP, and (where relevant) mScarlet fluorescence are shown (**A–C**). As illustrated in panel A, the broken gray lines show cell outlines as seen by DIC light microscopy. Scale bar is 5 µm. (**D**) Deconvoluted fluorescence signals from confocal microscopy of eGFP-ETP2 overexpressing cells (same cell line as in panel A). Individual channels as well as the overlay of signals from channels for Hoechst 33342, mScarlet, and eGFP are shown. (Upper row) Early stage in endosymbiont division, (lower row) later stage. Scale bar is 2 µm. Abbreviations: DIC, differential interference contrast; K, kinetoplast (network of concatenated mitochondrial DNA); N, nucleus; S, endosymbiont.

Previously, we observed that ETP2 fused at its N-terminus to the green fluorescent protein eGFP, overexpressed from the *δ-amastin* locus (Δδ*-ama^egfp-etp2^*), localizes at the ESDS (Fig. 1A and reference 18). To verify that eGFP-ETP2 expressed from its endogenous locus showed the same subcellular localization, we tagged ETP2 endogenously (Δ*etp2^egfp-etp2^*/*etp2*) in a parental strain expressing the endosymbiont marker mScarlet-ETP1 (Δ*γ-ama^mS-etp1^*; see reference 18) or in the wild-type (Wt) background. Epifluorescence microscopy revealed that in both cell lines, the endogenously tagged ETP2 showed the same localization as when overexpressed, although the eGFP fluorescence signal was weaker (Fig. 1B, micrographs 1 and 2). Cells in which both *etp2* alleles were replaced with *egfp-etp2* (Δ*etp2^egfp-etp2^*/Δ*etp2^egfp-etp2^*) showed an enhanced eGFP fluorescence signal at the ESDS (Fig. 1C; for confirmation of the cell line by PCR and Southern blot, see Fig. S2). Furthermore, no unusual phenotype was observed in these cells.

Confocal microscopic analysis revealed two different morphologies of the (overexpressed) fluorescent ETP2 signal. In cell cycle stage 1, during which the endosymbiont shows the typical peanut-shaped morphology and no cellular structures have duplicated yet, eGFP-ETP2 forms two patches at both sides of the endosymbiont envelope in optical transects of the ESDS (Fig. 1D, top). These patches appear to be part of a ring-shaped structure surrounding the ESDS as observed when moving through the Z-stacks (Movie S1). When cells move to cell cycle stage 2, defined by the bacterium becoming elongated and constricted in the middle, the two eGFP-ETP2 patches meet in the middle of the ESDS, slightly cupping the newly forming bacterial cell poles, resulting in an x-like structure (Fig. 1D, bottom, and Movie S2).

### Endosymbiont-encoded FtsZ and host-encoded ETP2 show a cell cycle-dependent localization at the ESDS

**Fig 2:**
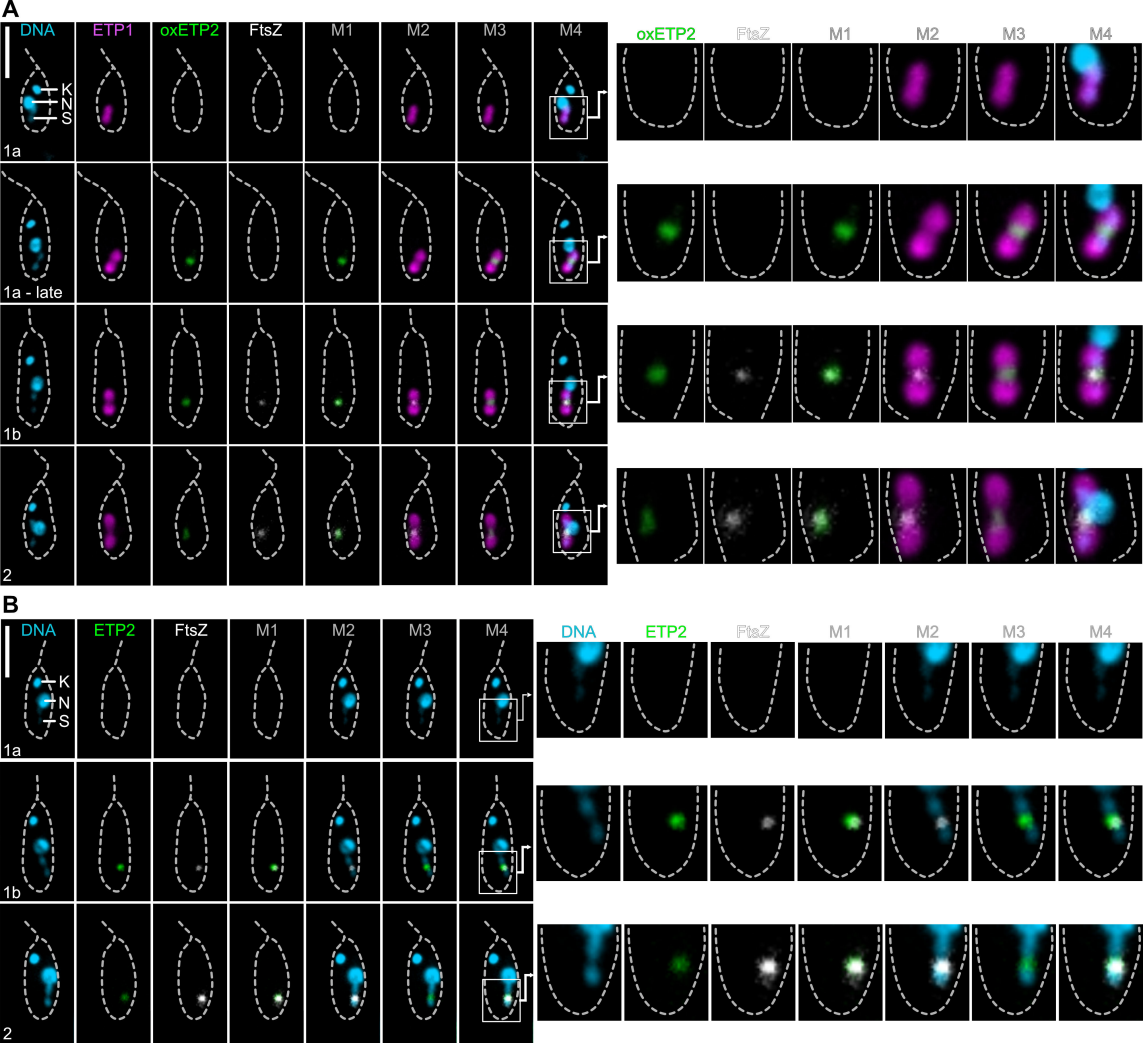
Subcellular localization of endosymbiont-encoded FtsZ and host-encoded recombinant ETP2 throughout early cell cycle stages. Subcellular localization of eGFP-ETP2 and FtsZ in mid-log phase cells co-expressing mScarlet-ETP1 (Δ*γ-ama^mS-etp1^*) and eGFP-ETP2 (overexpressed [Δ*δ-ama^egfp-etp2^*] in panel** A** and endogenously tagged [Δ*etp2^egfp-etp2^*/Δ*etp2^egfp-etp2^*] in panel** B** throughout early cell cycle stages as analyzed by epifluorescence microscopy. eGFP-ETP2 (green) and mScarlet-ETP1 (magenta) were detected by their autofluorescence, FtsZ (white) by IFA, and DNA (cyan) by Hoechst 33342 staining. Scale bars are 5 µm. Merges in panel A: Merge1 (M1), overlay of eGFP and FtsZ signals; Merge2 (M2), mScarlet and FtsZ; Merge3 (M3), mScarlet and eGFP; Merge4 (M4), all four channels. Merges in panel B: Merge1, overlay of eGFP and FtsZ signals; Merge2, FtsZ and Hoechst 33342; Merge3, eGFP and Hoechst 33342; Merge4, all three channels. Abbreviations are the same as in Fig. 1. Numbers on images show cell cycle stages compared to reference 9. White boxes in the left panels indicate areas of detailed images in the right panels.

Previously, reconstruction of cell cycle stages of *A. deanei* had shown that FtsZ and ETP9 exhibited a cell cycle-dependent localization at the ESDS (9). To test whether ETP2 displayed a similar localization dynamic, we visualized FtsZ by immunofluorescence assay (IFA) and eGFP-ETP2 and mScarlet-ETP1 by their autofluorescence and reconstructed division stages based on cell morphologies from images of >1,000 fixed cells (Fig. 2).

For the eGFP-ETP2 overexpressing cell line (Δ*δ-ama^egfp-etp2^*, Fig. 2A), initially neither FtsZ nor eGFP-ETP2 fluorescence signals were observed (stage 1a). Then, eGFP-ETP2 appears at the ESDS (late stage 1a). Arrival of FtsZ at the ESDS marks the beginning of stage 1b. Now, eGFP-ETP2 and FtsZ co-localize. During stage 2, the endosymbiont elongates and constricts with both FtsZ and ETP2 remaining co-localized at the ESDS. At later stages, the signal disappears in most cells, and only a few cells show diffuse or inconsistent fluorescence signals for both proteins. For the endogenously tagged ETP2 (Δ*etp2^egfp-etp2^*/Δ*etp2^egfp-etp2^*, Fig. 2B), the localization patterns appear similar; however, it seems that both proteins, eGFP-ETP2 and FtsZ, reach the ESDS at a similar time point. Hence, the arrival of the overexpressed eGFP-ETP2 at the ESDS before FtsZ might rather reflect its better detectability than actual early arrival, and a clear order of arrival cannot be unambiguously established.

### Homozygous *etp2* deletion mutant is viable and shows severe division phenotypes

To study the cellular function of ETP2, we initially generated a heterozygous *etp2* deletion mutant in which one allele of *etp2* was replaced by the neomycin phosphotransferase gene, *neo^R^* (Δ*etp2^neo^*/*etp2*, Fig. S2). This mutant showed no noticeable phenotype. Several attempts to replace the remaining *etp2* allele by a hygromycin phosphotransferase gene, *hyg^R^*, or a phleomycin-binding protein, *phleo^R^*, failed. However, we obtained a homozygous deletion mutant, in which the second allele was disrupted by the insertion of a *hyg^R^*-containing cassette into the *etp2* open reading frame, resulting in a heavily truncated ETP2 protein (Δ*etp2^neo^*/Δ*etp2_246-491_^hyg^*, Fig. S2). This mutant clone grew up after 10 days (typically clones are obtained up to 7 dasys post-limiting dilution) and was the only positive clone from two attempts to disrupt *etp2*, in which 15 clones were obtained in total, with the remaining ones being PCR negative and, hence, likely carried insertions into other loci. Both heterozygous and homozygous *etp2* deletion mutants were generated not only in the symbiotic but also in an aposymbiotic *A. deanei* strain (ATCC PRA-265 and ATCC 30969, respectively) (for verification, see Fig. S2).

**Fig 3:**
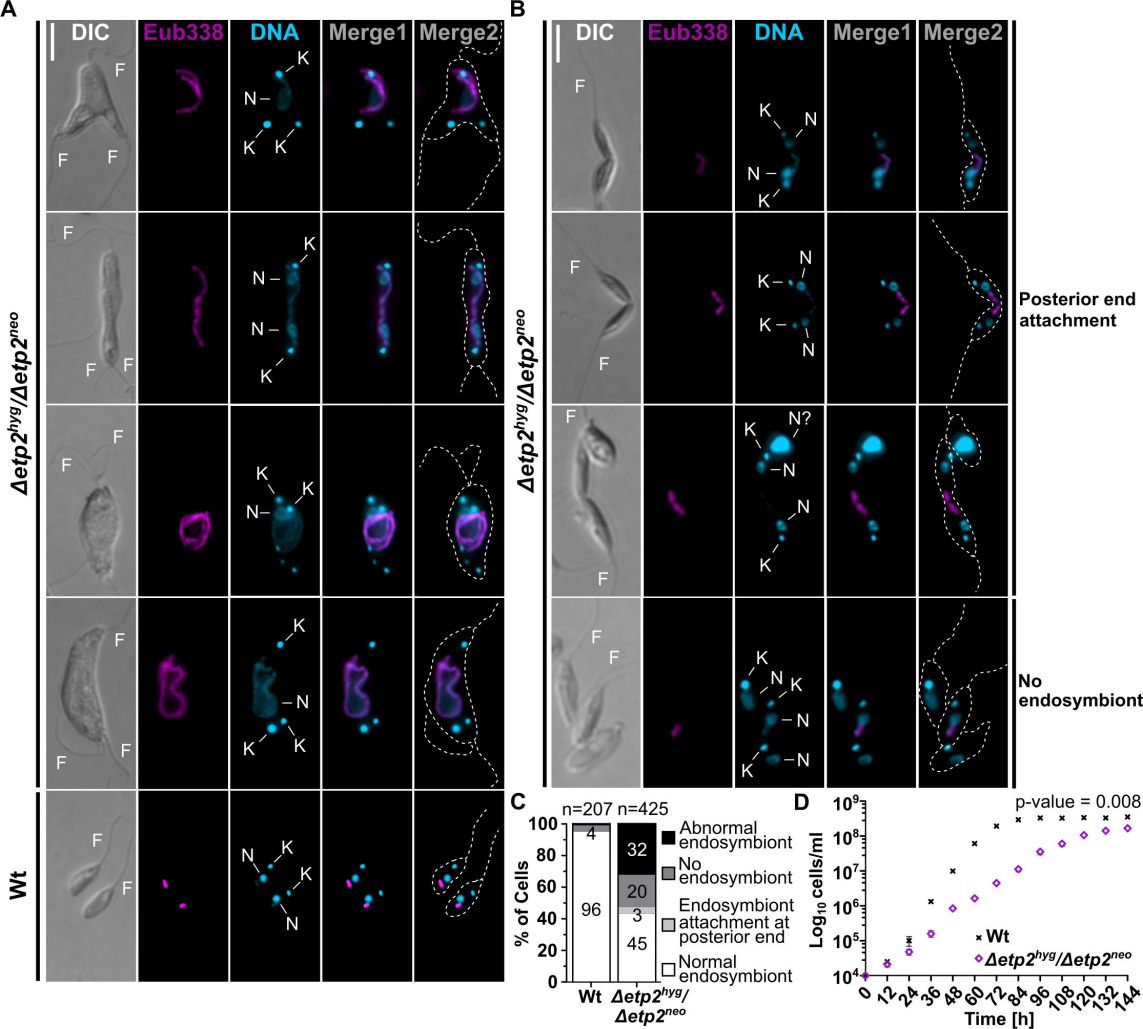
Characterization of homozygous *etp2* deletion mutants in symbiotic *A. deanei*. (**A and B**) Fluorescence *in situ* hybridization (FISH) micrographs of homozygous *etp2* mutant and Wt cells from mid-log phase. Merge1, superposition of signals from Hoechst 33342-stained DNA (cyan) and the Cy3-Eub338 FISH probe directed against the bacterial 16S rRNA (magenta). Merge2, superposition of Hoechst 33342 and Cy3 signals on cell outlines as seen by DIC (broken gray lines). Abbreviations: F, flagellum; K, kinetoplast; N, nucleus. Scale bar is 5 µm. (A) Filamentous endosymbionts in distorted host cells are featured. (B) “Morphologically normal” host cells during the final stages of cytokinesis, attached by an undivided endosymbiont or cell with one morphotypical endosymbiont or cells that lost their endosymbiont. For overview pictures, see Fig. S3. (**C**) Quantification of division phenotypes in the *etp2* mutant compared to Wt cells by microscopic analysis of 425 and 207 randomly chosen cells, respectively. (**D**) Growth curves of Wt and *etp2* deletion mutant cells. Data represent mean ± SD of five biological replicates. Area under the curve (AUC) was calculated for each replicate based on log-transformed cell counts over time. AUC values were compared between strains using a Wilcoxon rank-sum exact test to assess differences in overall growth dynamics (*P* = 0.007937).

Interestingly, in the symbiotic homozygous *etp2* deletion mutant, 32% of the cells displayed long, filamentous endosymbionts (Fig. 3A, “abnormal” in Fig. 3C) accompanied by severely distorted host cells; 20% of the cells lost their endosymbiont (Fig. 3B and C; for overview pictures, see Fig. S3). Distorted host cells displayed multiple kinetoplasts and flagella and, notably, a singular and enlarged nucleus in most cases, suggesting that both kinetoplast and nuclear DNA replication remain active, yet only segregation of the kinetoplast is fulfilled. Intriguingly, 3% of host cells appeared in the final stages of cytokinesis, attached only by an apparently undivided endosymbiont localized at the posterior end of the cells (Fig. 3B and C; Fig. S3, white arrowhead). Additionally, some cells lacking endosymbionts were noted in the final stages of cytokinesis (Fig. S3, black arrowhead) and possibly completing it without fulfilling complete segregation of the endosymbiont. However, 45% of the cells appeared “normal,” retaining a singular, morphotypical endosymbiont (Fig. 3B and C). Hence, the disruption of the second *etp2* allele is not entirely lethal, suggesting that although now error prone, a successful endosymbiont division is still possible. This is reflected also by the significantly slower growth of the *etp2* deletion mutant compared to Wt cells (Fig. 3D). In the aposymbiotic homozygous *etp2* deletion mutant, cells did not differ morphologically from Wt cells (Fig. S4), suggesting that the function of ETP2 is confined toward the endosymbiont.

### KD of ETP2 results in elongated endosymbionts in severely distorted host cells

**Fig 4:**
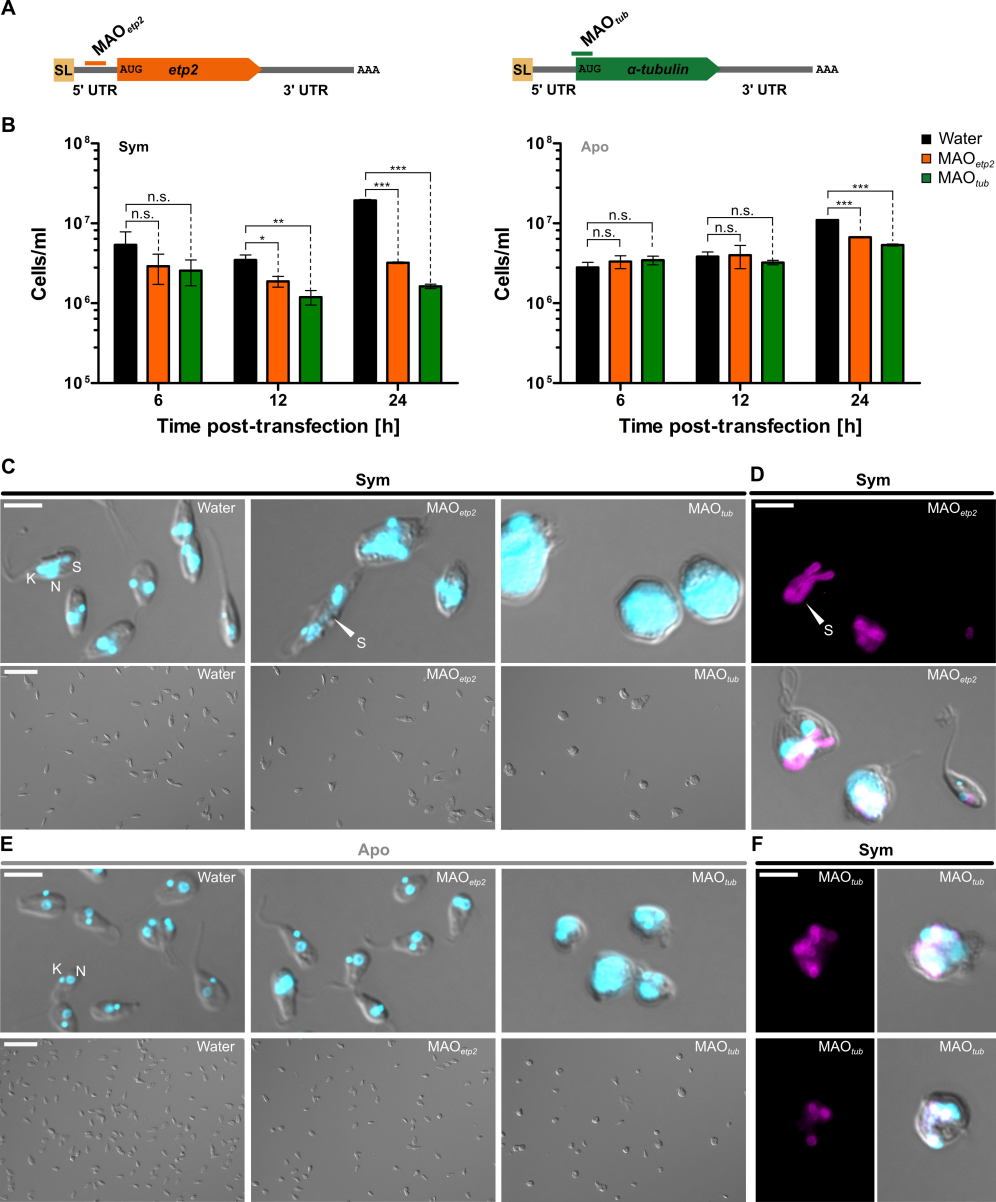
KD of ETP2 in symbiotic and aposymbiotic *A. deanei* strains. (**A**) Schematic representation of MAO binding sites on the target mRNA of *etp2* and *α-tubulin*. (**B**) Cell densities of cultures of the symbiotic and aposymbiotic *A. deanei* strains 6, 12, and 24 h post-transfection with water, MAO*_etp2_*, or MAO*_tub_*. Plotted are mean and standard deviation from three technical replicates. Statistical differences were examined by unpaired Student’s *t*-test. Symbols denote statistical significance. *, *P* < 0.05; **, *P* < 0.01; ***, *P* < 0.001; two-tailed. (**C**) Micrographs of the symbiotic cells 24 h post-transfection for each treatment. A detail micrograph (merge of DIC and Hoechst 33342, upper panel) and overview micrograph (DIC, lower panel) are shown. (**D**) Micrographs of symbiotic cells expressing the endosymbiont marker mScarlet-ETP1 24 h post-transfection with MAO*_etp2_*. The mScarlet channel alone (upper panel) and merge of DIC, Hoechst 33342, and mScarlet channels (lower panel) are shown. For overview images, see Fig. S5A. (**E**) Micrographs of aposymbiotic cells 24 h post-transfection for each treatment. A detailed micrograph (merge of DIC and Hoechst 33342, upper panel) and an overview micrograph (DIC, lower panel) are shown. (**F**) Micrographs of symbiotic cells expressing the endosymbiont marker 24 h post-transfection with MAO*_tub_*. The mScarlet channel alone (left panel) and merge of DIC, Hoechst 33342, and mScarlet channels (right panel) are shown. Abbreviations: K, kinetoplast; N, nucleus; S, symbiont; SL, spliced leader; UTR, untranslated region. Arrowhead highlights the symbiont. Scale bars are 5 µm (for detail) and 25 µm (for overview micrographs).

To confirm independently that the observed division phenotype in *etp2* deletion mutants is caused by the lack of ETP2, we used a KD approach based on morpholino antisense oligos (MAOs). For this, we transfected symbiotic and aposymbiotic *A. deanei* Wt cells with MAOs against *etp2* mRNA (MAO*_etp2_*) (Fig. 4A). Hybridization of MAOs with the 5′ UTR of a specific mRNA results in the repression of its translation (20). Consistent with the phenotype observed in the *etp2* deletion mutants (Fig. 3), 24 h post-transfection, we observed a strong reduction in growth and the formation of long, filamentous endosymbionts in distorted host cells in the symbiotic strain (Fig. 4B and C), whereas in the aposymbiotic strain, no noticeable changes in cell morphology were observed (Fig. 4E). However, a slight but significant growth reduction was observed in the aposymbiotic strain following transfection with MAO*_etp2_* compared to the water control, which might reflect off-target effects (Fig. 4B). For better visualization of the endosymbionts, cells expressing the endosymbiont marker mScarlet-ETP1 were also transfected with MAO*_etp2_*, resulting in the same filamentous endosymbionts (Fig. 4D; for overview images, see Fig. S5A).

Importantly, symbiotic and aposymbiotic cells mock-treated with water showed the highest cell numbers and no aberrant cell morphologies 24 h post-transfection (Fig. 4B, C and E), while cells transfected with MAO*_tub_* against α-tubulin (Fig. 4A), as a positive control, exhibited the expected effects (Fig. 4B, C and E), with a markedly reduced growth and formation of roundish cell morphologies known from *Trypanosoma brucei* α-tubulin KDs (21). Furthermore, a massive accumulation of DNA-containing compartments was observed following α-tubulin KD. This phenomenon was more pronounced in the symbiotic strain, probably due to its larger cell and organelle sizes, in addition to the presence of the endosymbiont (see Fig. S6). The mScarlet-ETP1 signal shows that although clumps of endosymbionts appear in the division-impaired α-tubulin-depleted host cells, the endosymbionts appeared to be fully divided and not filamentous, suggesting that endosymbiont division itself was not impaired (Fig. 4F).

### Z-ring formation is independent of ETP2; recruitment of ETP9 to the ESDS depends on ETP2

**Fig 5:**
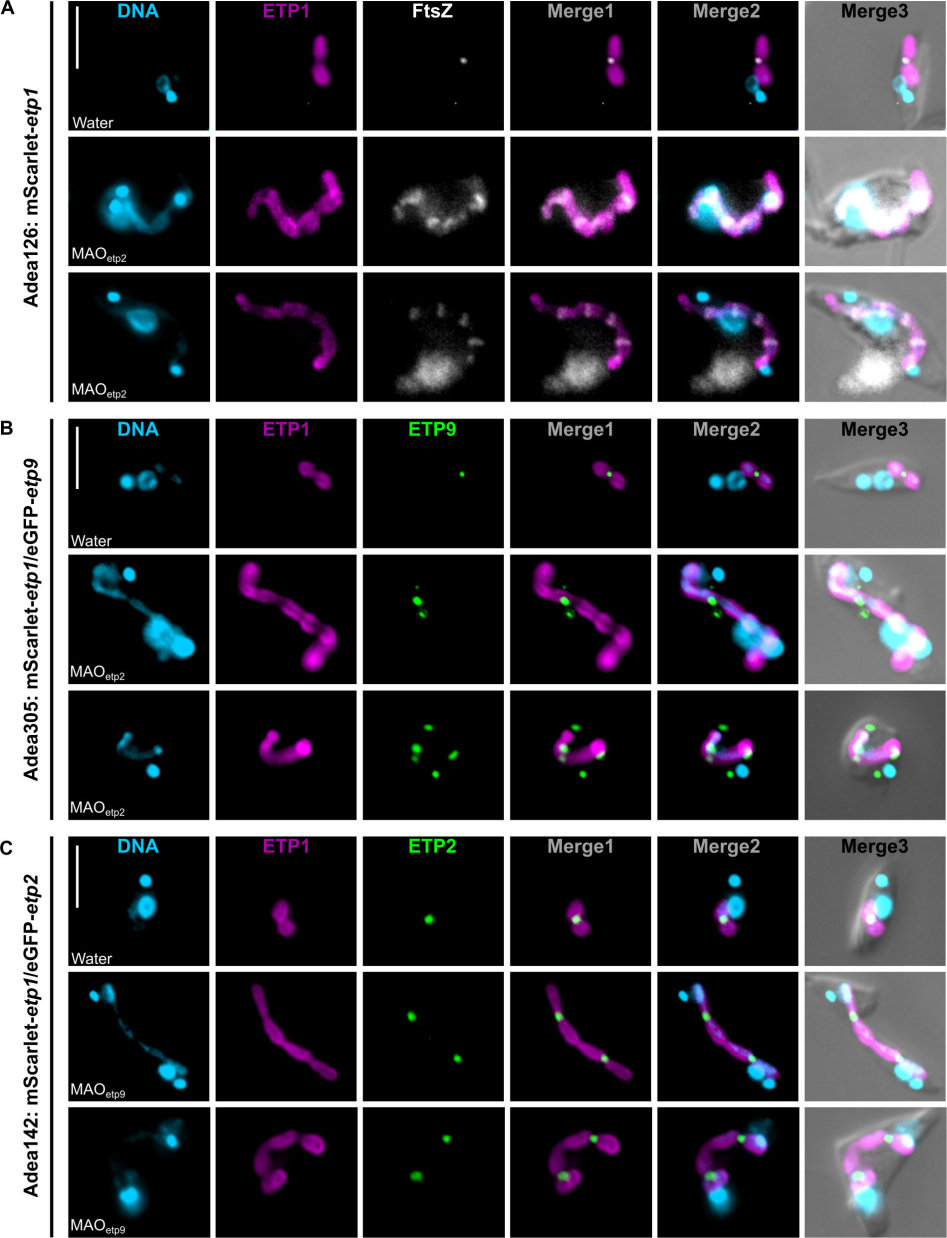
Interdependence of the subcellular localization of FtsZ, ETP2, and ETP9 in *A. deanei*. (**A**) Localization of the bacterium-encoded FtsZ in cells expressing the endosymbiont marker mScarlet-ETP1 24 h post-transfection with MAO*_etp2_*. (**B**) Localization of eGFP-ETP9 in cells co-expressing eGFP-ETP9 and mScarlet-ETP1 24 h post-transfection with MAO*_etp2_*. For overview images, see Fig. S5B. (**C**) Localization of eGFP-ETP2 in cells co-expressing eGFP-ETP2 and mScarlet-ETP1 24 h post-transfection with MAO*_etp9-1_* (against *etp9* [9]). For overview images, see Fig. S5C. Shown are the fluorescence signals of Hoechst 33342-stained DNA (cyan), mScarlet-ETP1 autofluorescence (magenta), eGFP (green), and the IFA signal for FtsZ (white). Merge1, overlay of fluorescence signals of mScarlet-ETP1 and FtsZ or eGFP; Merge2, DNA, mScarlet-ETP1, and FtsZ or eGFP; Merge3, DNA, mScarlet-ETP1, FtsZ or eGFP, and the DIC picture. In all three panels, corresponding cells mock-treated with water instead of the MAO are shown as a control. Scale bar is 5 µm.

To assess the effects of ETP2 depletion on the localization of FtsZ, we repeated the ETP2 KD (in cells expressing mScarlet-ETP1) followed by IFA using an antibody raised against FtsZ. Twenty-four hours post-transfection, FtsZ was observed to form several foci along the filamentous endosymbionts (Fig. 5A), suggesting that Z-ring formation is independent of ETP2.

Next, we repeated the ETP2 KD in cells co-expressing eGFP-ETP9 and mScarlet-ETP1. Intriguingly, when ETP2 is depleted, ETP9 forms several foci in the host cell, many at or in the proximity of the endosymbiont (Fig. 5B; Fig. S5B), but does not form the typical ring structures around the ESDS anymore (9). The reverse experiment, ETP9 KD in cells co-expressing eGFP-ETP2 and mScarlet-ETP1, resulted in the formation of several ETP2 rings around the division-impaired filamentous endosymbiont (Fig. 5C; Fig. S5C), similar to the distribution of FtsZ in ETP9 KD cells (9). These results demonstrate that recruitment of ETP9 to the ESDS depends on the presence of ETP2, whereas ETP2 recruitment is independent of ETP9.

### ETP2, ETP7, and ETP9 are exclusively found within the Strigomonadinae, and ETP2 is likely an intrinsically disordered protein

**Fig 6:**
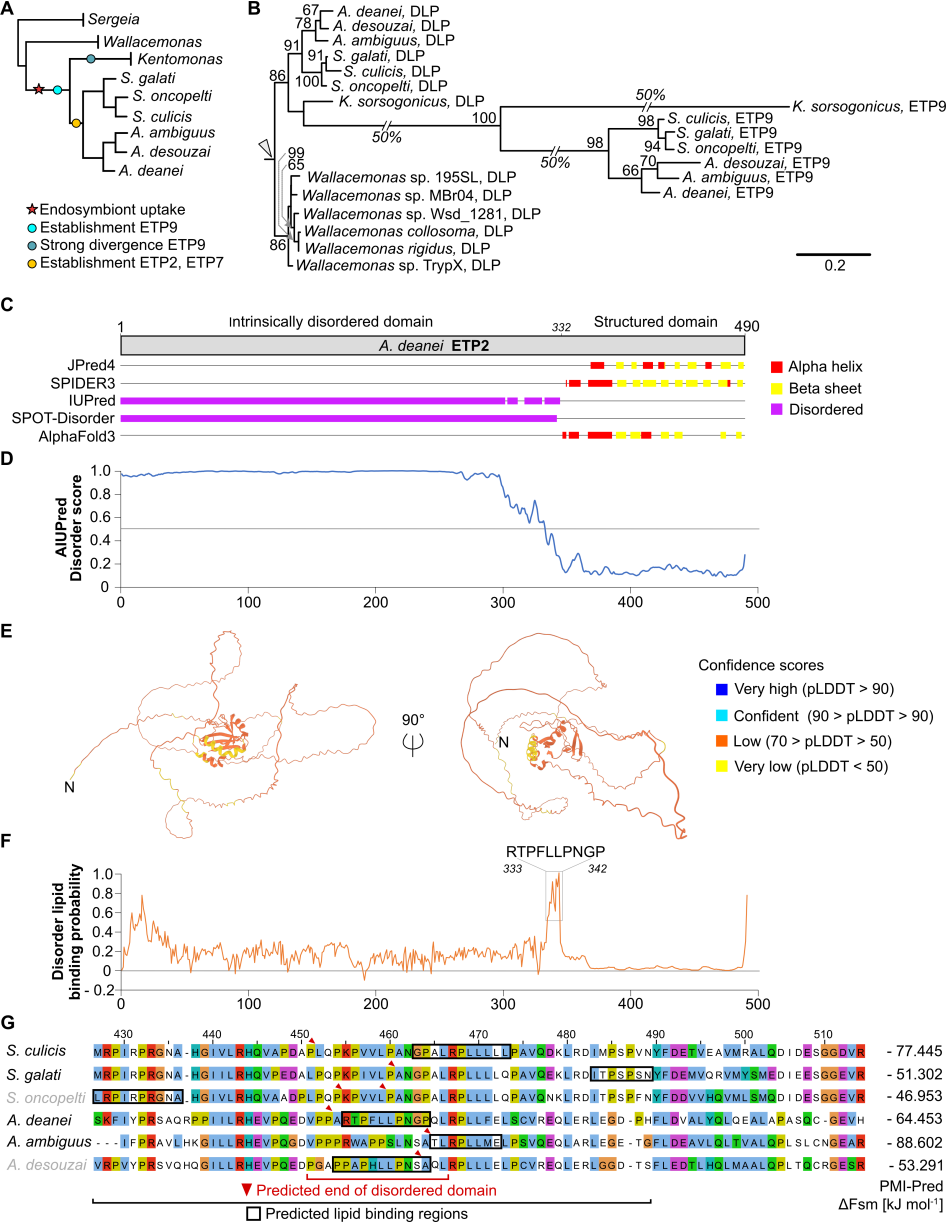
*In silico* analyses predict ETP2 as an intrinsically disordered protein with putative lipid-binding capability. (**A**) Schematic tree representing the phylogenetic relationship of the taxa tested for the presence of ETP orthologs by tBlastN (for phylogeny and genome availability, see references 23 and 24). Symbols on the tree indicate endosymbiont and ETP acquisition or divergence (as indicated). (**B**) Maximum likelihood analysis of dynamin-like proteins in Strigomonadinae and *Wallacemonas*. Very long branches were reduced in length as indicated. Arrowhead, root; values at branches, bootstrap support >50%. (**C**) Overview of secondary structure elements and intrinsically disordered domains of ETP2 predicted by JPred4, SPIDER3, IUPred, SPOT-Disorder, and AlphaFold3 (as indicated). Consensus of the start of the structured domain of ETP2 at amino acid position 332 is indicated. (**D**) Results of AIUPred analysis of ETP2 using the default options (AIUPred, only disorder, default smoothing). Prediction scores of >0.5 (above gray line) indicate disordered regions. (**E**) AlphaFold3-predicted tertiary structure of ETP2. The N-terminus of the protein is marked with “N.” Confidence scores are indicated on the right. (**F**) Results of DisoLipPred analysis of ETP2. Larger scores denote higher likelihood of a disordered lipid-binding region. The region containing the highest likelihood (amino acids 333–342) is highlighted. (**G**) ClusterO alignment of the interface region between the unstructured and structured domains of ETP2 orthologs. Predicted disordered lipid-binding regions from DisoLipPred are highlighted with black boxes, and the red arrows indicate the start of the structured region as predicted by AIUPred. These regions are well conserved across orthologs, although the precise position of the predicted lipid-binding motifs varies slightly. Predicted free energies for membrane binding of the highlighted sequences, calculated using PMI-Pred with the “negatively charged membrane” option, are shown on the right. Free energy values (*ΔFsm*) less than –28 kJ/mol indicate a high likelihood of membrane binding. Species name in gray indicates incomplete sequence. For more details, see Fig. S9.

ETP9 likely arose in the Strigomonadinae by duplication and divergence from a pre-existing DLP involved in mitochondrion division and endocytosis (18). To gain a deeper understanding of the origin of ETP2 and ETP7, we investigated their distribution by Basic Local Alignment Search Tool (BLAST) searches. BlastP against the National Center for Biotechnology Information (NCBI) nr and EukProt (22) databases yielded hits exclusively to *A. deanei* and *Strigomonas culicis* (for ETP2) or *A. deanei* alone (for ETP7), suggesting that their genes arose in members of the Strigomonadinae. However, the Strigomonadinae are represented in these databases only by these two species, and their closest known relatives without endosymbiont, the genera *Wallacemonas* and *Sergeia*, are not represented at all. Hence, we extended our searches using tBlastN against draft genome assemblies available on NCBI for Strigomonadinae, *Wallacemonas*, and *Sergeia* spp. These extended searches revealed that ETP2 and ETP7 are present throughout *Angomonas* and *Strigomonas* spp. but missing in *Kentomonas*, represented only by *Kentomonas sorsogonicus* (Fig. 6A). The C-terminal part in both proteins is relatively conserved (85%–26% identity over alignment positions 427 to end for ETP2; and 88%–43% identity over positions 580 to end for ETP7); the N-terminal part is very divergent between *Angomonas* and *Strigomonas* spp. (Fig. S7).

By repeating this more fine-grained distribution analysis also for ETP9, we found ETP9 to be present throughout all Strigomonadinae members, including *K. sorsogonicus*, but missing in *Wallacemonas* and *Sergeia*, which contain only the regular trypanosomatid DLP (Fig. 6A and B), suggesting that ETP9 arose in the common ancestor of the Strigomonadinae. We have shown before that *A. deanei* ETP9 apparently retained the typical DLP domain organization and structure, despite low sequence conservation compared to the typical trypanosomatid DLPs (9). The ETP9 ortholog in *K. sorsogonicus*, however, diverged so much that it is unclear whether it can still adopt the classical DLP fold (Fig. S8).

To gain insights into the domain organization of ETP2, we analyzed its sequence for the presence of known domains using HHPred (25) and SMART (26); however, no significant hits were identified. Secondary structure prediction tools, including JPred4 (27) and SPIDER3 (28), predicted the presence of alpha helices and beta sheets exclusively within the conserved C-terminal region, beyond amino acid position 343 (Fig. 6C; Fig. S7A). In accord with the absence of predicted structural motifs, intrinsic disorder was predicted by IUPred (29), SPOT-Disorder (30), and AIUPred (31) in the N-terminal region up to amino acid position 332 (Fig. 6C and D). Consistent with these analyses, AlphaFold3 (32) predicted for ETP2 a generally structured C-terminus and unstructured N-terminus (Fig. 6E). However, the predicted tertiary structure exhibited low to very low predicted local distance difference test scores across the entire protein.

The observed localization pattern of recombinant ETP2 (Fig. 1) suggests that the protein interacts at the ESDS with the (outer or inner) endosymbiont membrane, and we identified a region (*A. deanei* ETP2_333–342_) that shows high probability of harboring a disordered lipid-binding motif, as predicted by DisoLipPred (33) (Fig. 6F). Notably, a disordered lipid-binding motif in this region is predicted across all ETP2 orthologs (Fig. 6G; Fig. S9). The potential membrane-binding capability of these motifs was further supported by PMI-Pred (34) analyses, which predicted free energy levels of −47 to −89 kJ/mol for binding of these motifs to a negatively charged membrane, well below the required threshold of −28 kJ/mol, supporting membrane binding (34).

## DISCUSSION

*A. deanei* has been recently reported to manifest nuclear control over its endosymbiont’s division by means of the DLP ETP9 that apparently forms a contractile ring structure around the ESDS (9). Here, we functionally characterized a second nucleus-encoded protein, ETP2, previously reported to localize at the ESDS (18).

We found that recombinant ETP2 localizes at the ESDS specifically at cell cycle stages in which the endosymbiont divides (Fig. 1 and 2). Replacement of both *etp2* alleles with the recombinant copy without a resulting phenotype (Fig. 1C) demonstrated that its N-terminal fusion to eGFP did not affect the localization or function of ETP2. The accumulation of ETP2 at the ESDS appears to precede the emergence of ETP9, which arrives at the ESDS after Z-ring formation (compare Fig. 2 and reference 9). The functional involvement of ETP2 in endosymbiont division is demonstrated by the striking division phenotype exhibited by *A. deanei* cells following *etp2* deletion or KD, with filamentous endosymbionts in highly distorted host cells containing multiple kinetoplasts and flagella (Fig. 3A through C and 4). We witnessed cases where non-distorted host cells undergoing cytokinesis appeared to remain attached through their posterior ends by an undivided endosymbiont (Fig. 3B; Fig. S3, white arrowhead) and apparently successfully detaching host cells not inheriting an endosymbiont (Fig. S3, black arrowhead), likely giving rise to distorted host cells with filamentous endosymbionts as well as aposymbiotic cells. We have shown before that, in strain ATCC PRA-265, aposymbiotic cells do not survive (9).

Since FtsZ localizes in distinct foci along the filamentous endosymbionts following ETP2 KD (Fig. 5A), recruitment of FtsZ to the prospective ESDS, which is likely controlled by the endosymbiont-encoded Min system (9), is apparently unaffected by ETP2 depletion. Conversely, ETP9 mis-localizes following ETP2 KD (Fig. 5B), demonstrating that ETP2 is involved in recruiting ETP9 to the ESDS. However, the fact that the *etp2* deletion cell line remains alive yet exhibits slower growth than the Wt (Fig. 3D) lends to the conclusion that despite the disruption of the last *etp2* allele, in a subset of the population, ETP9 still finds its way to the ESDS, enabling endosymbiont division and regular host cell cytokinesis downstream. Whether this outcome depends on the presence of the truncated ETP2_1–245_, which remains in the homozygous deletion mutant (Fig. S2D) and might still have residual capability to direct ETP9 to the ESDS, or ETP9 itself has a limited ability to associate with the ESDS is currently unclear. In fact, some ETP9 foci in ETP2-depleted cells appear to be tightly associated with the endosymbiont envelope, suggesting an intrinsic membrane-binding capacity of ETP9. Interestingly, in *K. sorsogonicus* that contains a very divergent ETP9 ortholog (Fig. 6A and B; Fig. S8), ETP2 is missing, suggesting that *Ks*ETP9 either does not function in endosymbiont division or finds the ESDS independently or with the help of a different adaptor protein that evolved in this species.

In aposymbiotic *A. deanei* cells, ETP2 deletion or KD does not cause a division phenotype (Fig. S4; Fig. 4E), demonstrating that the ETP2 function is specific for endosymbiont division and the observed distorted host morphology is a secondary effect resulting from impaired endosymbiont division. The reduced growth of aposymbiotic cells following ETP2 KD (Fig. 4B) may represent an off-target effect. However, in the short 5′ UTR of *etp2,* no suitable binding site for a non-overlapping MAO was found that would help to verify this interpretation.

Our BLAST, hidden Markov modeling, and SMART analyses demonstrated that no ETP2 orthologs or potential domains therein are found in organisms outside of the Strigomonadinae. Secondary and tertiary structure predictions for ETP2 suggested an unstructured N-terminal domain and a structured C-terminus, albeit with low confidence scores for tertiary structure prediction (Fig. 6C through E). Collectively, these findings point to an origin of ETP2 via either *de novo* innovation from non-coding DNA or extensive sequence divergence beyond recognition within the Strigomonadinae (35), mechanisms that may underline its intrinsically disordered nature (36). Intrinsically disordered proteins often transiently interact with multiple binding partners and, thus, act in the form of signaling hubs or scaffolds as temporal organizers (37–41).

In sum, (i) the early arrival of ETP2 at the ESDS, (ii) the severe division phenotypes resulting from its disruption or depletion, (iii) the mis-localization of ETP9 in ETP2-depleted cells, and (iv) its distribution over *Angomonas* and *Strigomonas* that correlates with the distribution of the typical (i.e., not highly divergent) ETP9 indicate a function of ETP2 in the recruitment of ETP9 to the ESDS. Recruitment of the DLP ETP9 is essential for endosymbiont division. This function of ETP2 is supported by its predicted lipid-binding site, which is conserved across ETP2 orthologs and extended disordered regions that might support a function as a scaffold or signaling hub. What recruits ETP2 to the ESDS is currently unclear. Possible features that may play a role are negative membrane curvature or so far unknown interaction partners that interact, across membranes, with the bacterial Z-ring. Hence, although our findings lend further credence to the idea that the endosymbiont division machinery in *A. deanei* is of dual genetic origin, the full characterization of this fascinating structure remains in its infancy.

## MATERIALS AND METHODS

### Microbial strains, media, and growth conditions

Symbiotic *A. deanei* (ATCC PRA-265), aposymbiotic *A. deanei* (ATCC 30969), and *Escherichia coli* TOP10 cells, used for plasmid preparation, were grown as described previously (9).

### Plasmid generation

Primers used in this study are listed in Table S1. Schematic maps of plasmids and strains are displayed in Fig. S10.

For the generation of homozygous *etp2* deletion mutants, plasmid pAdea457 was generated. For this, the pUMA1467 backbone (42) was amplified from plasmid pAdea369 using the primer pair 3237/3244. The first half and second half of the *etp2* gene were used as 5′ and 3′ flanking region (fr) and were amplified from *A. deanei* genomic DNA (gDNA) using primer pairs 3238/3239 and 3242/3243, respectively. A fragment containing the *γ-amastin* 5′ fr with its spliced leader donor sequence, *hyg^r^*, and the intergenic region between the *gapdh1* and *gapdh2* genes, gapdh ir, was amplified from pAdea368 using primer pair 3240/3241. Fragments were assembled by Gibson cloning (43).

For the generation of pAdea115 (used for endogenous tagging of the first *etp2* allele), the fragment *etp2* fr 3′-pUMA1467-*etp2* fr 5′ was amplified from plasmid pAdea092 using primer pair 1015/1016, and the fragment *neo^r^-*gapdh ir-*egfp-etp2* was amplified from pAdea035 using primer pair 1022/1023. Both fragments were ligated by Golden Gate cloning (44).

For the generation of pAdea477 (used for endogenous tagging of the second *etp2* allele), primer pair 3322/3323 was used for amplification of the entire pAdea115 template except *neo^r^* and primer pair 3320/3321 to amplify *hyg^r^* from pAdea260. Both fragments were ligated by Gibson cloning.

All plasmids were amplified in *E. coli* TOP10 and isolated by NucleoSpin Plasmid kit (Macherey-Nagel). Correct assembly was verified by sequencing (Microsynth AG, Balgach, Switzerland).

### Transfection of *A. deanei* and verification of transgenic cell lines by PCR

Plasmid DNA (~40 µg) was digested with restriction enzyme(s) cutting at the 5′ and 3′ ends of the insertion cassette (Fig. S10). Linearized cassettes were transfected into *A. deanei*, and clones were selected and verified by PCR as described earlier (18). Primers that were used (Table S1) either bind in the genomic region outside of the inserted cassette or one primer binding outside and the other inside the cassette. Only verified strains were used for further analysis.

### Southern blot analysis

For the replacement of the second *etp2* allele, insertion of a single cassette was verified by Southern blot analysis as described before (18). Briefly, ~7.5 µg of digested gDNA was resolved and transferred to a nylon membrane. A digoxigenin-labeled probe against *hyg^r^* (*α-hyg^r^*; see Fig. S2) was generated (for primers, see Table S1) and hybridized to the immobilized DNA at 53°C. Lastly, the membrane was developed as per the protocol supplied with the DIG-High Prime DNA Labeling and Detection Starter kit II (Roche Applied Science). The resulting chemiluminescence signal was detected by a Chemidoc MP (Bio-Rad).

### Epifluorescence microscopy

Epifluorescence microscopy was performed as described before (18). In brief, cells in the mid-log phase were fixed with formaldehyde, spotted onto poly-L-lysine-coated glass slides, stained with Hoechst 33342, and coated with antifade reagent SlowFade Diamond (Thermo Fisher Scientific). Images were acquired with an Axio Imager M.1 (Zeiss, Oberkochen, Germany) using an EC Plan-Neofluar ×40/1.3 oil Ph3 or ×100/1.30 Oil Ph3M27 objective (Zeiss). Images were analyzed with Zen Blue v.2.5 and processed with ImageJ v.2.0 software.

### IFA

FtsZ was visualized by IFA as described before (9). In brief, fixed cells immobilized on glass slides were permeabilized with 0.2% vol/vol Triton-X100, blocked, and incubated with anti-FtsZ_pepC_ primary antibody (raised in rabbit against *Ca*. K. crithidii FtsZ; see reference 9). Next, cells were washed and incubated with anti-rabbit IgG (goat, polyclonal, Abberior Star-red, Ex: 638 nm) secondary antibody before Hoechst 33342 staining, covered with antifade reagent, and imaged (as above). mScarlet-ETP1 and eGFP-ETP2 were detected based on their autofluorescence. For the reconstruction of cell cycle stages, more than 1,000 cells were analyzed.

### Fluorescence *in situ* hybridization

For visualizing the endosymbionts by fluorescence *in situ* hybridization (FISH), cells were fixed (for 30 min) and immobilized on glass slides (as above). Glass slides were air-dried for 30 min; cells were dehydrated in rising concentrations of ethanol (50%, 80%, and 100%) for 3 min each. Then, 5 µL of a 5′ Cy3-labeled Eub338 probe (50 ng DNA/µL) directed against the bacterial 16S rRNA (45, 46) was mixed with 45 µL of hybridization buffer (900 mM NaCl, 20 mM Tris-HCl, pH 7.2, 35% formamide, and 10% SDS in dH_2_O) and spotted onto the dry slides. The slides were incubated at 46°C for 2 h in a humid chamber. Afterward, the slides were washed in FISH-wash buffer (80 mM NaCl, 20 mM Tris-HCl, pH 7.2, and 10% SDS in dH_2_O) and incubated at 48°C for 25 min. The slides were rinsed twice with dH_2_O. Finally, cells were stained with Hoechst 33342, covered with antifade reagent, and imaged (as above).

### Confocal microscopy

Slides were prepared as described earlier (9). Imaging was performed at an inverted confocal microscope, Leica TCS SP8 (STED 3X), with standard settings as described before (18), except that the laser line was set at 35% for eGFP and 15% for mScarlet. For the deconvolution of the raw data, Huygens Professional v.23.10.0p7 64b was used with default settings except that in manual mode, a threshold for background extraction was set based on background fluorescence signals. Generation of supplementary movies was performed as described earlier (9).

### KD assays using MAOs

For KDs, *A. deanei* cells were transfected with MAOs as described earlier (9) using the new MAO*_etp2_* (5′-CGTAGTCCATTTTGGTGTGTATGAT-3′) and previously described MAO*_etp9-1_* and MAO*_tub_*, all synthesized by Gene Tools, LLC, Philomath, OR, USA. After transfection, cells were transferred into brain heart infusion (BHI) medium supplemented with 10 µg/mL hemin and 10% vol/vol horse serum (both from Sigma Aldrich) without antibiotics for all strains and incubated at 28°C. Samples were collected at 6, 12, and 24 h post-transfection and used for Hoechst 33342 staining and epifluorescence microscopy as described before (9).

### Growth measurements

To compare growth of the homozygous *etp2* mutant and Wt cell lines, 10^4^ cells/mL were inoculated in 20 mL BHI medium supplemented with hemin without antibiotics and incubated at 28°C (five replicates per cell line). Cells were counted every 12 h over 144 h using a cell counter (Multisizer 4e, Beckman Coulter). Growth curves were summarized by calculating the area under the curve (AUC) for each of five biological replicates per strain, resulting in a single interpretable metric of overall growth performance (47). Differences in AUC between strains were evaluated using the Wilcoxon rank-sum exact test. For MAO-treated cells, counting was performed in triplicate at 6, 12, and 24 h post-transfection, and differences between treatments were compared by Student’s *t*-test.

### *A. deanei* cell and organelle measurements

For measuring the width and length of symbiotic and aposymbiotic *A. deanei* strains, cell line Adea126 expressing mScarlet-ETP1 and Wt aposymbiotic strains were used, respectively. For width and length measurements of the cell, differential interference contrast images were used. For kinetoplast and nucleus, Hoechst 33342 staining was used. For measuring the endosymbiont, mScarlet-ETP1, localized at the bacterial envelope, was used. Measurements were performed manually with Zen v.2.5 on cells that were categorized into different cell cycle stages. Mean values were calculated and plotted using GraphPad Prism v.5.0.

### Analysis of ETP distribution and phylogeny

BLASTp searches (*E*-value threshold 1.0E-6) were performed against the NCBI nr and EukProt (22) databases. Additionally, whole genome shotgun sequences from six Strigomonadinae species, six *Wallacemonas* spp., and *Sergeia podlipaevii* available on NCBI (https://www.ncbi.nlm.nih.gov/datasets/genome/) were downloaded. For species names, strain, and accession numbers see Table S2. In these assemblies, encoded proteins homologous to ETPs were identified by tBlastN using *A. deanei* ETP2, ETP7, and ETP9 as a query (*E*-value cutoff 1.0E-6). Genome regions generating best blast hits were translated, and the obtained protein sequences were subjected to reverse BlastP against the *A. deanei* translated proteome. Bidirectional best blast hits were regarded as orthologous proteins. Multiple sequence alignments of homologous proteins were generated with Clustal Omega (48).

For phylogenetic analysis of ETP9, DLP protein sequences obtained from Strigomonadinae and *Wallacemonas* spp. as an outgroup were aligned (as above). Well-aligned sequence blocks were extracted, resulting in 436 aligned positions, and phylogeny was inferred by maximum likelihood analysis using IQ-TREE v.2.2.079 (49) with automatic model selection. Branch support was estimated from 100 non-parametric bootstrap replicates.

## SUPPLEMENTAL MATERIAL

Supplemental material mbio.02247-25-s0001.pdf Supplemental figures, tables, and movie legends.

Movie S1 mbio.02247-25-s0002.avi Cell co-expressing eGFP-ETP2 and mScarlet-ETP1 at an early stage of endosymbiont division.

Movie S2 mbio.02247-25-s0003.avi Cell co-expressing eGFP-ETP2 and mScarlet-ETP1 at a later stage of endosymbiont division.

ASM does not own the copyrights to Supplemental Material that may be linked to, or accessed through, an article. The authors have granted ASM a non-exclusive, world-wide license to publish the Supplemental Material files. Please contact the corresponding author directly for reuse.
