# Supplementary material for: Host-encoded ETP2 is involved in recruiting the dynamin-like protein ETP9 to the endosymbiont division site in trypanosomatid Angomonas deanei
Source: mBio. 2025 Sep 12;16(10):e02247-25. doi: 10.1128/mbio.02247-25 (PMC12505957; doi:10.1128/mbio.02247-25)
Supplement: Supplemental material — Supplemental figures, tables, and movie legends. [file mbio.02247-25-s0001.pdf]

Supplementary Materials

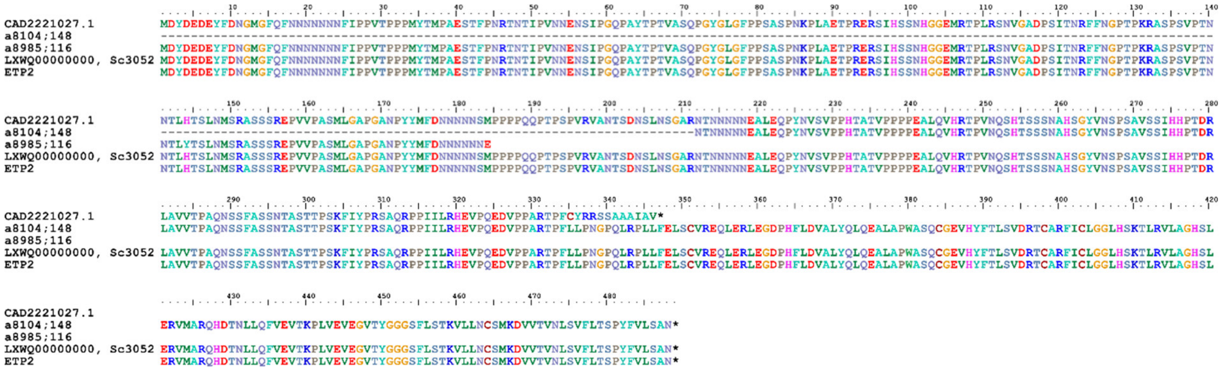

Figure S1: ETP2 sequence. Comparing *A. deanei* protein CAD2221027.1 [from the chromosome-level nuclear genome assembly GCA_903995115.1 (NCBI accession)] to our transcriptome data (represented by two partial transcripts a8104;148 and a8985;116) and scaffold 3052 [from our scaffold-level nuclear genome assembly GCA_001659865.1 (NCBI accession)] (see Morales et al. 2016) allowed for correction of the ETP2 amino acid sequence.

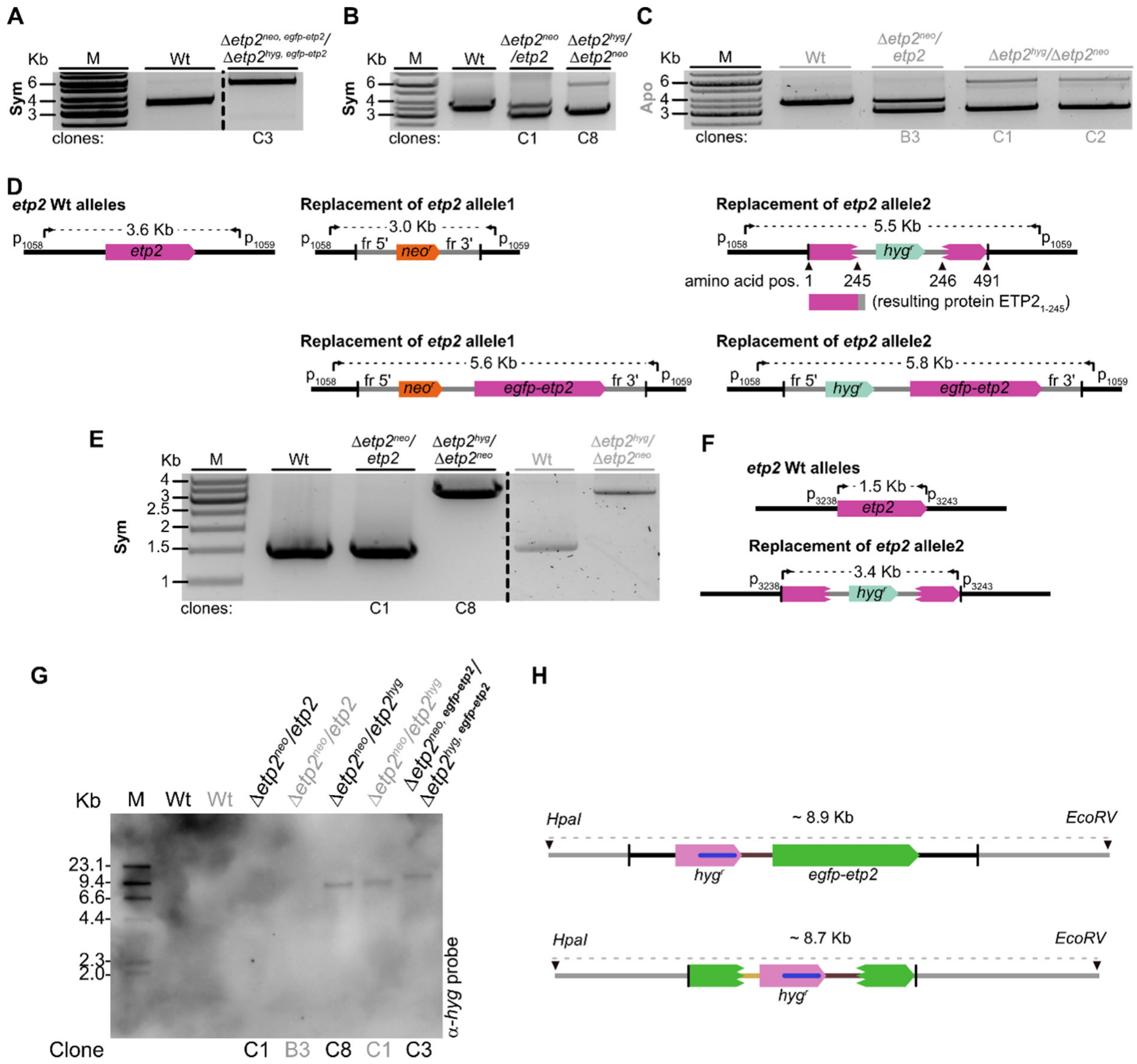

Figure S2: Verification of recombinant *A. deanei* cell lines by PCR and Southern blot analysis. A: Verification of the symbiotic strain with both alleles of *etp2* replaced by an *egfp-etp2*-containing cassette. **B:** Verification of symbiotic *etp2* heterozygous and homozygous deletion mutant cell lines. **C:** Verification of aposymbiotic heterozygous and homozygous *etp2* deletion mutant cell lines. **D:** Maps of Wt and modified *etp2* loci with expected band sizes detected by PCR (for A-C). Black arrowhead marks the amino acid position. **E:** PCR analysis of presence/absence of an intact *etp2* copy in symbiotic and aposymbiotic *A. deanei* Wt and *etp2* deletion mutant cell lines. **F:** Maps of Wt and modified *etp2* locus with expected band sizes detected by PCR in E. Black text shows symbiotic and grey aposymbiotic strains. Dotted lines show expected PCR band sizes. Curved arrows show primer binding sites. Vertical black lines on the maps show borders of inserted DNA fragments in the genome. Broken pink/green boxes on the maps show first and second halves of the *etp2* ORF used as fr 5' and fr 3' for homologous recombination, respectively (in D, F, H). **G:** Southern blot verification of *A. deanei* strains with Wt and modified *etp2* loci using a probe against *hyg*^{r}. M, DIG-labelled DNA marker. **H:** Maps of Wt and recombinant *etp2* loci analyzed by Southern blot. Black arrowheads on the maps indicate the cutting site for the indicated restriction enzymes. The grey dotted lines indicate the expected size of the digested fragment. Binding sites of the α-*hyg* probe are marked by a blue bar. Abbreviations: fr, flanking region; *hyg*^{r}, hygromycin resistance gene, and *neo*^{r}, neomycin resistance gene.

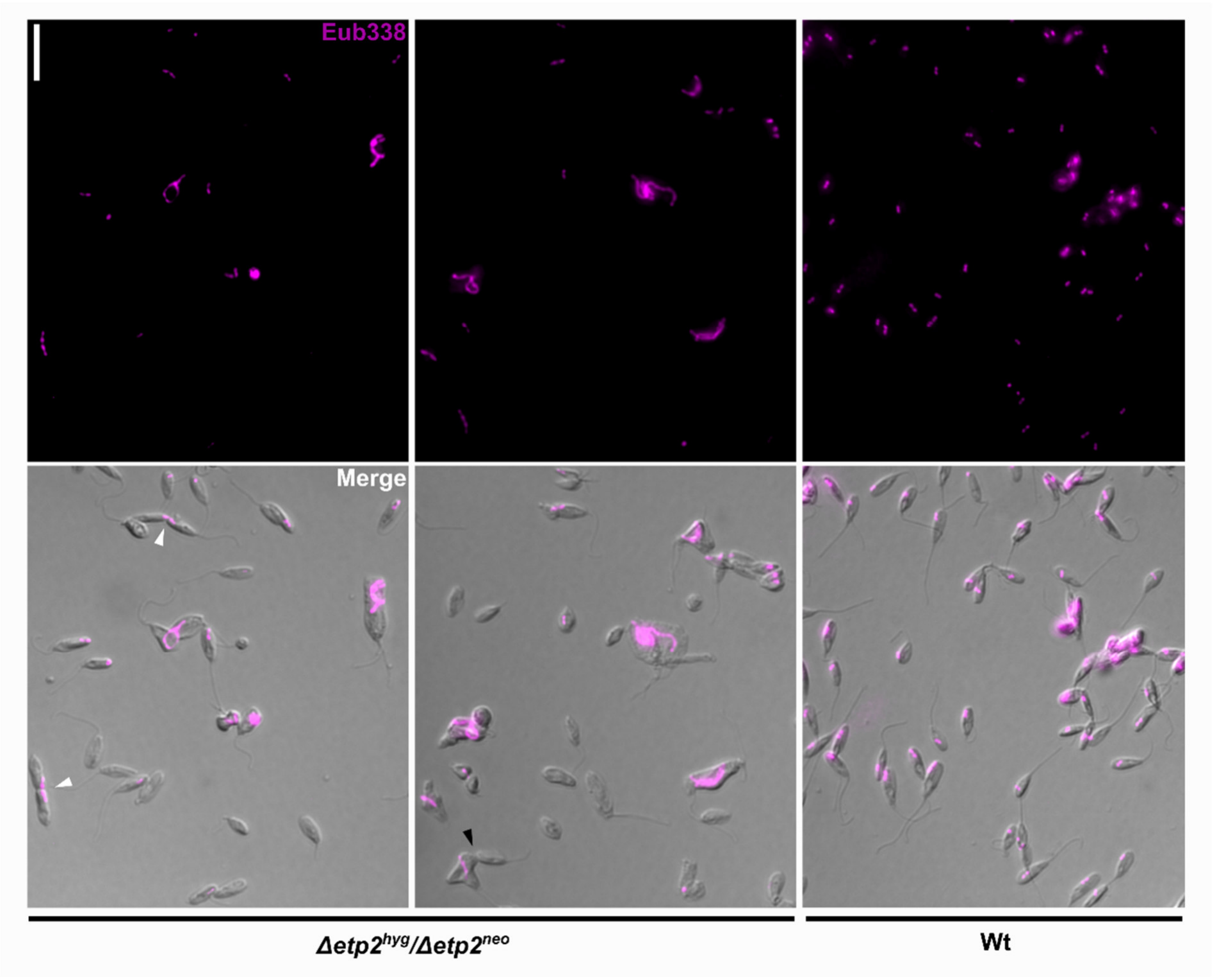

Figure S3: Overview micrographs of cells shown in Fig. 3. Cy3-Eub338 FISH-stained homozygous *etp2* mutant cells show the occurrence of cells with abnormal, normal, or lacking endosymbionts. White arrowheads indicate host cells during the final stages of cytokinesis, however, attached by an undivided endosymbiont at the posterior end. Black arrowhead indicates a host cell in the final stage of cytokinesis lacking an endosymbiont. Cy3-Eub338 FISH-stained Wt cells are shown as a control. Scale bar is 10 µm. Upper row shows the Cy3 fluorescence signal in magenta, lower row superposition of Cy3 signal and DIC image.

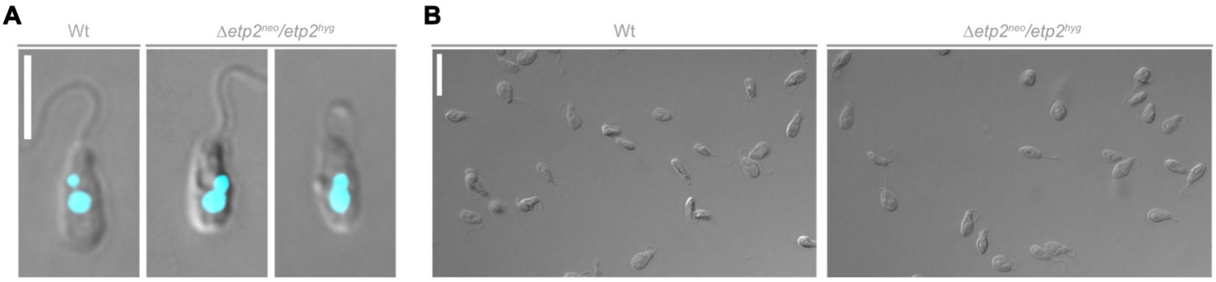

Figure S4: Effects of deletion of both alleles of *etp2* in the aposymbiotic *A. deanei* strain. A: The figure shows representative images of Wt and the homozygous *etp2* deletion mutant cell line in the aposymbiotic *A. deanei* strain. Images show an overlay of DIC and Hoechst 33342 fluorescence (cyan). Scale bar is 5 µm. **B:** Overview DIC images of cells of the same cultures. Scale bar: 10 µm. Grey text/bars indicate aposymbiotic strain.

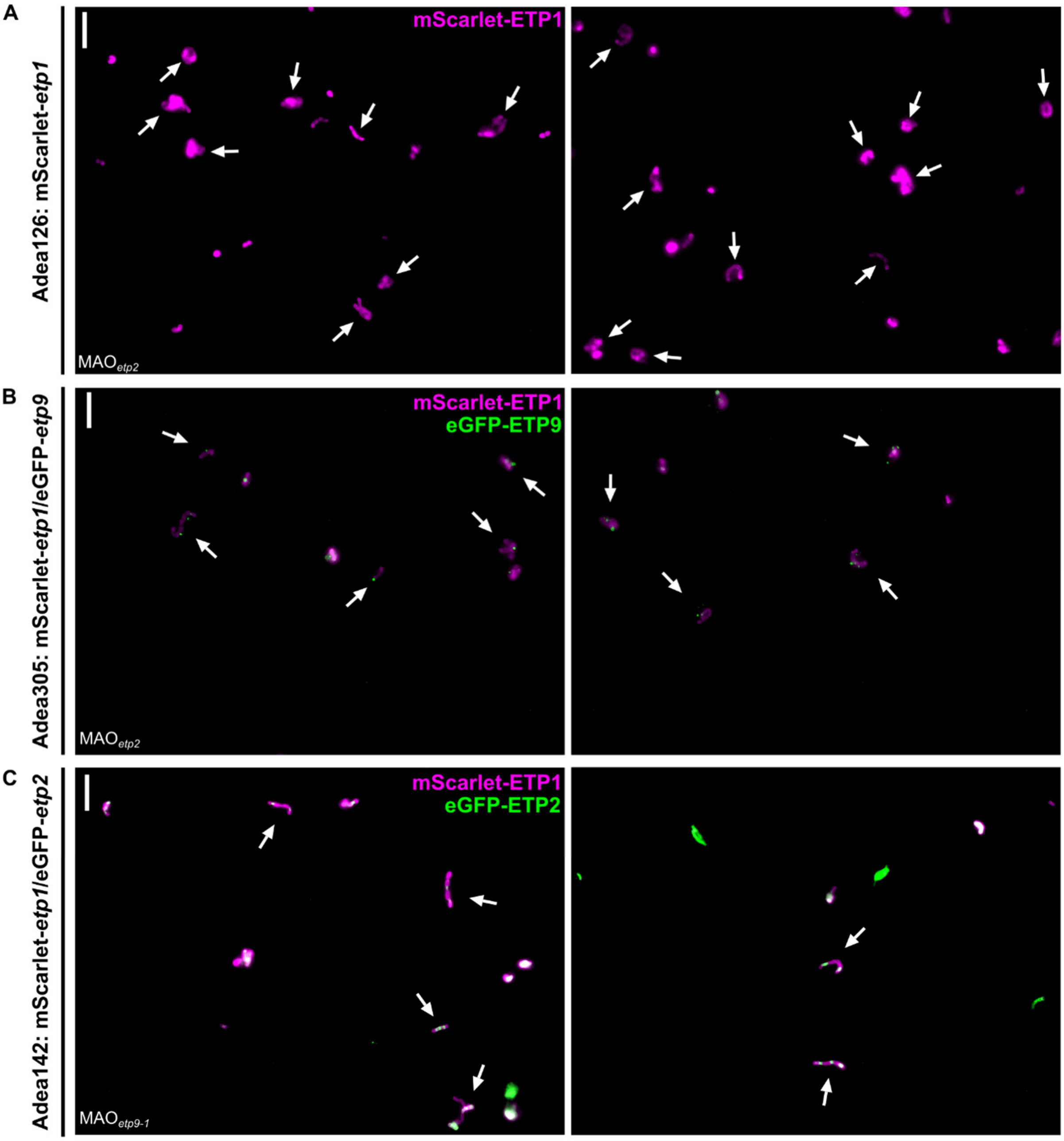

Figure S5: Overview pictures showing the effects of KD of ETP2 or ETP9. A: *A. deanei* cells expressing the endosymbiont marker mScarlet-ETP1 24 h post-transfection with MAO$_{etp2}$. **B:** *A. deanei* cells co-expressing the endosymbiont marker mScarlet-ETP1 with eGFP-ETP9 24 h post-transfection with MAO$_{etp2}$. **C:** *A. deanei* cells co-expressing the endosymbiont marker mScarlet-ETP1 with eGFP-ETP2 24 h post-transfection with MAO$_{etp9-1}$. Shown are the mScarlet channel alone (A) or a merge of the mScarlet and eGFP channels (B, C). Arrows highlight filamentous endosymbionts (A) and filamentous endosymbionts in which the localization pattern of the eGFP-tagged ETPs are clearly visible (B, C). Scale bars are 10 µm.

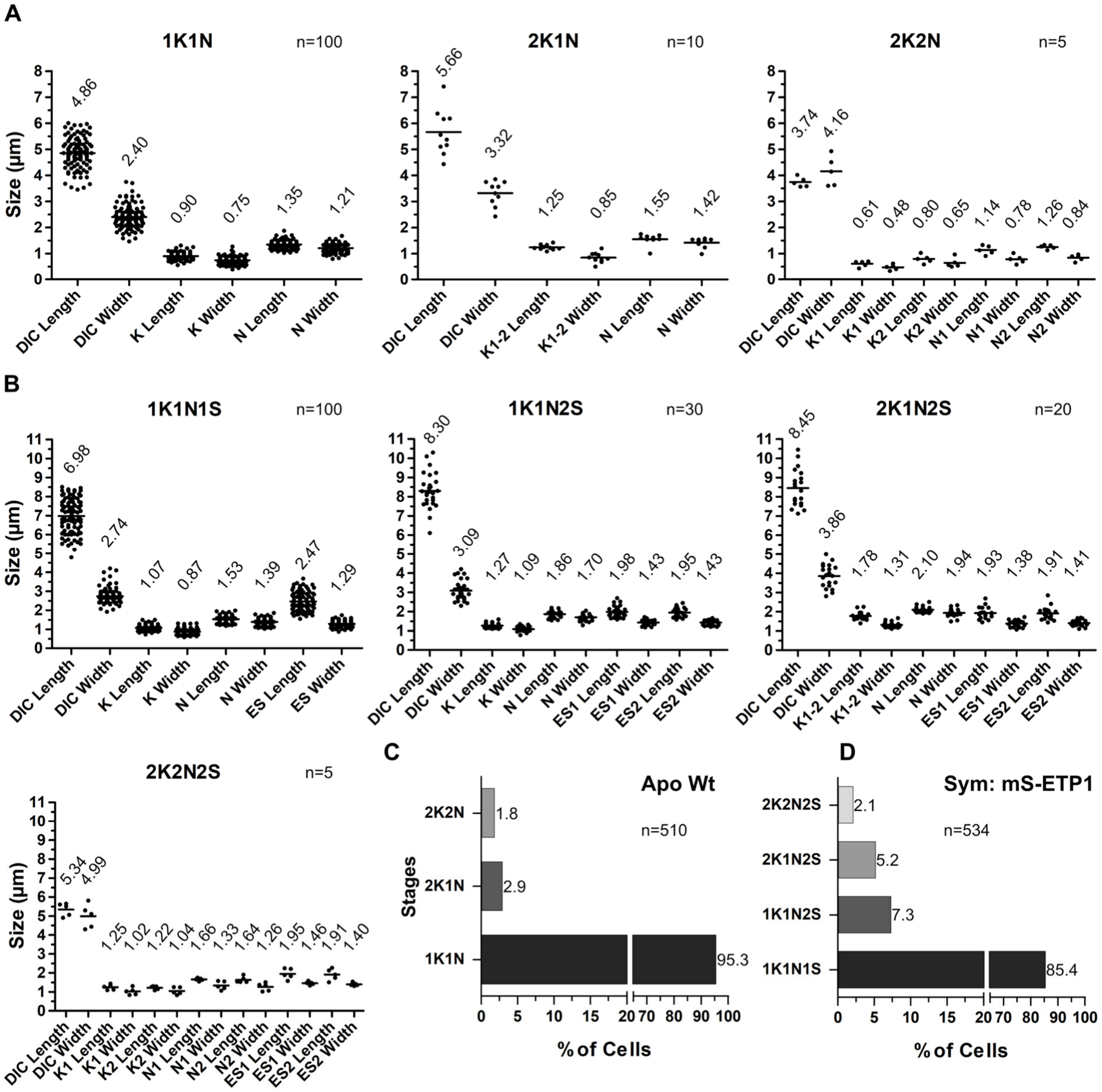

Figure S6: Measurements of cell and organelle sizes for symbiotic and aposymbiotic strains. A: Measurements for aposymbiotic Wt cells at different cell cycle stages- 1K1N, 2K1N, and 2K2N (see below). **B:** Measurements for symbiotic cells expressing the endosymbiont marker mScarlet-ETP1 at different cell cycle stages- 1K1N1S, 1K1N2S, 2K1N2S, and 2K2N2S (see below). Mean values are represented on each plot for each measurement by a horizontal line. DIC images were used to measure cell size, Hoechst 33342-stained DNA for kinetoplast (K) and nucleus (N), and mScarlet-ETP1-labelled endosymbiont (ES) for bacterium measurements, respectively. K1-2 represents two kinetoplasts that are still not completely separated. **C:** Quantification of approximately 500 aposymbiotic Wt cells categorized into different cell cycle stages. **D:** Quantification of approximately 500 symbiotic cells expressing mScarlet-ETP1 categorized into different cell cycle stages. Cell cycle stages are described by the number of kinetoplasts (K), nuclei (N), and endosymbionts (S) per cell.

A

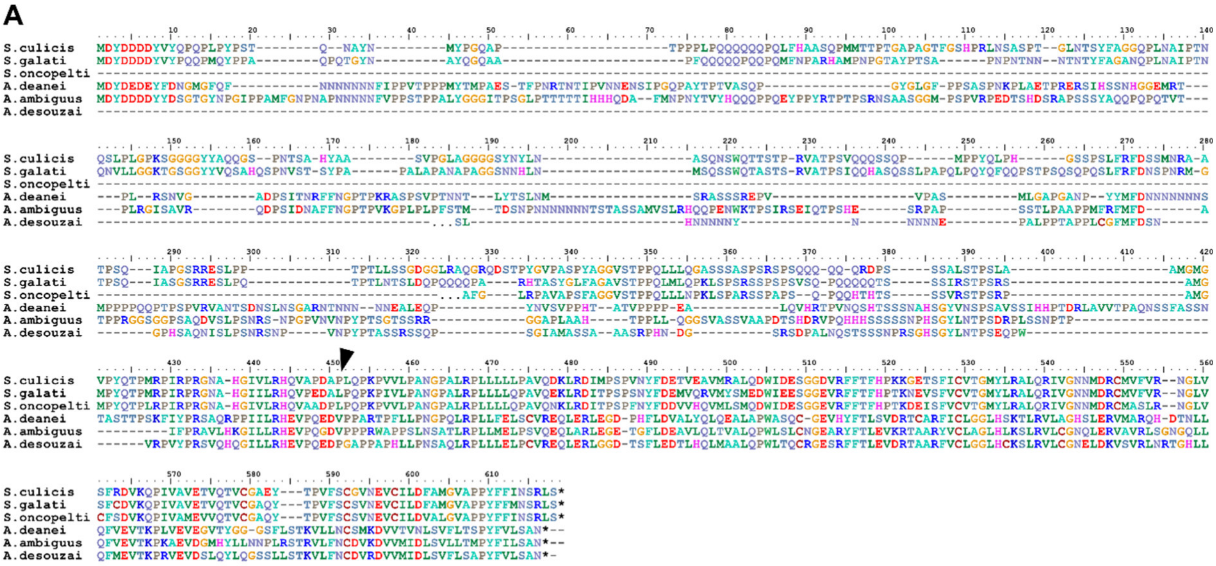

B

% $ID_{427-END}$	A.dea	A.des	A.amb	S.gal	S.cul	S.onc
A.deanei	-	60.85	56.61	26.70	25.65	25.65
A.desouzai		-	64.02	29.69	30.73	30.21
A.ambiguus			-	28.13	28.13	27.08
S.galati				-	85.48	85.48
S.culicis					-	82.80

C

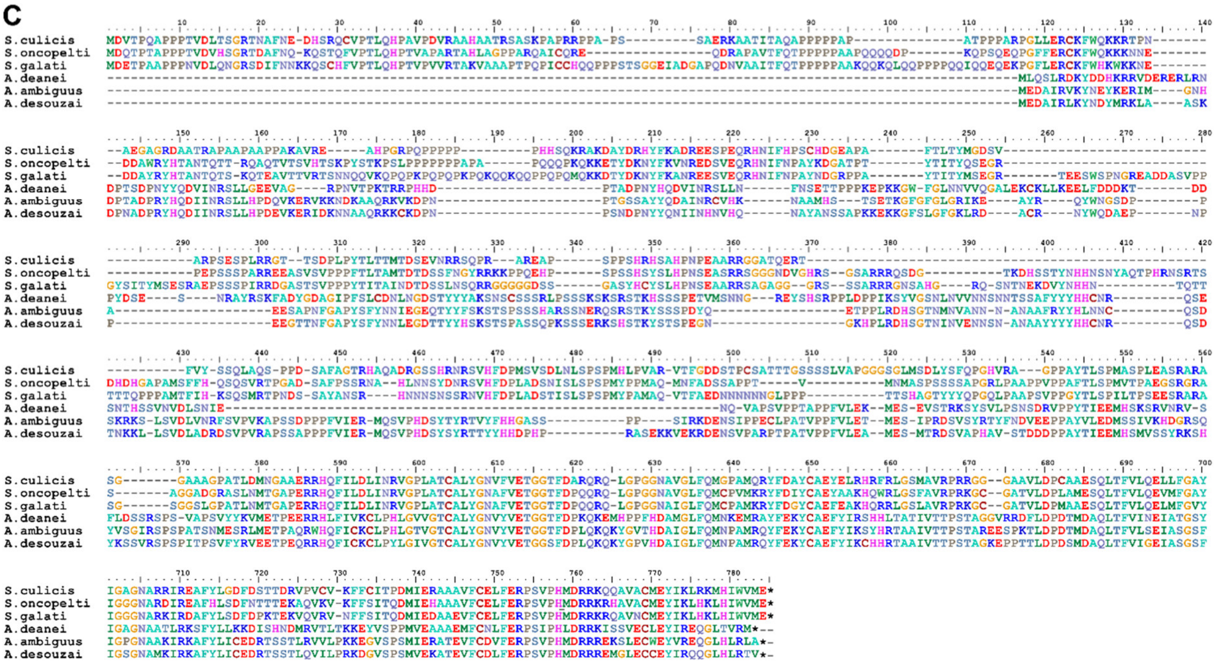

D

% $ID_{580-END}$	A.dea	A.des	A.amb	S.gal	S.cul	S.onc
A.deanei	-	66.18	67.16	42.93	43.90	41.95
A.desouzai		-	83.82	47.80	45.37	45.37
A.ambiguus			-	64.83	46.34	44.88
S.galati				-	80.10	88.06
S.culicis					-	80.10

Figure S7: Conservation of ETP2 and ETP7 throughout the Strigomonadinae. Displayed are ClustalO alignments (A, C) and protein identity matrices (B, D) of the conserved C-terminal part of ETP2 and ETP7, respectively. **A:** Note, that sequences for *S. oncopelti* and *A. desouzai* ETP2 are incomplete as their genes span contig breaks. The black arrowhead indicates amino acid position 332 of *A. deanei* ETP2 that marks the transition from the unstructured to the structured domain. Sequence identities: *S. culicis*, AUXH01000165.1: 14091-12643; *S. oncopelti*, AUXK01006954.1: 2-757; *S. galati*, AUXN01000928.1: 3252-4715; *A. ambiguus*, JADNSB010000028.1: 168835-167252; *A. desouzai*, AUXL01005260.1: 2-910. **B:** The matrix provides amino acid identity between ETP2 orthologs over

alignment positions 427 to 619 (in A). **C:** Sequence identities: *S. culicis*, AUXH01000109.1: 39672-37867; *S. oncopelti*, AUXK01000200.1: 6795-8747; *S. galati*, AUXN01000027.1: 25586-23463; *A. ambiguus*, JADNSB010000070.1: 39901-41550; *A. desouzai*, AUXL01000051.1: 8957-10630. **D:** The matrix provides amino acid identity between ETP7 orthologs over alignment positions 580 to 784 (in C).

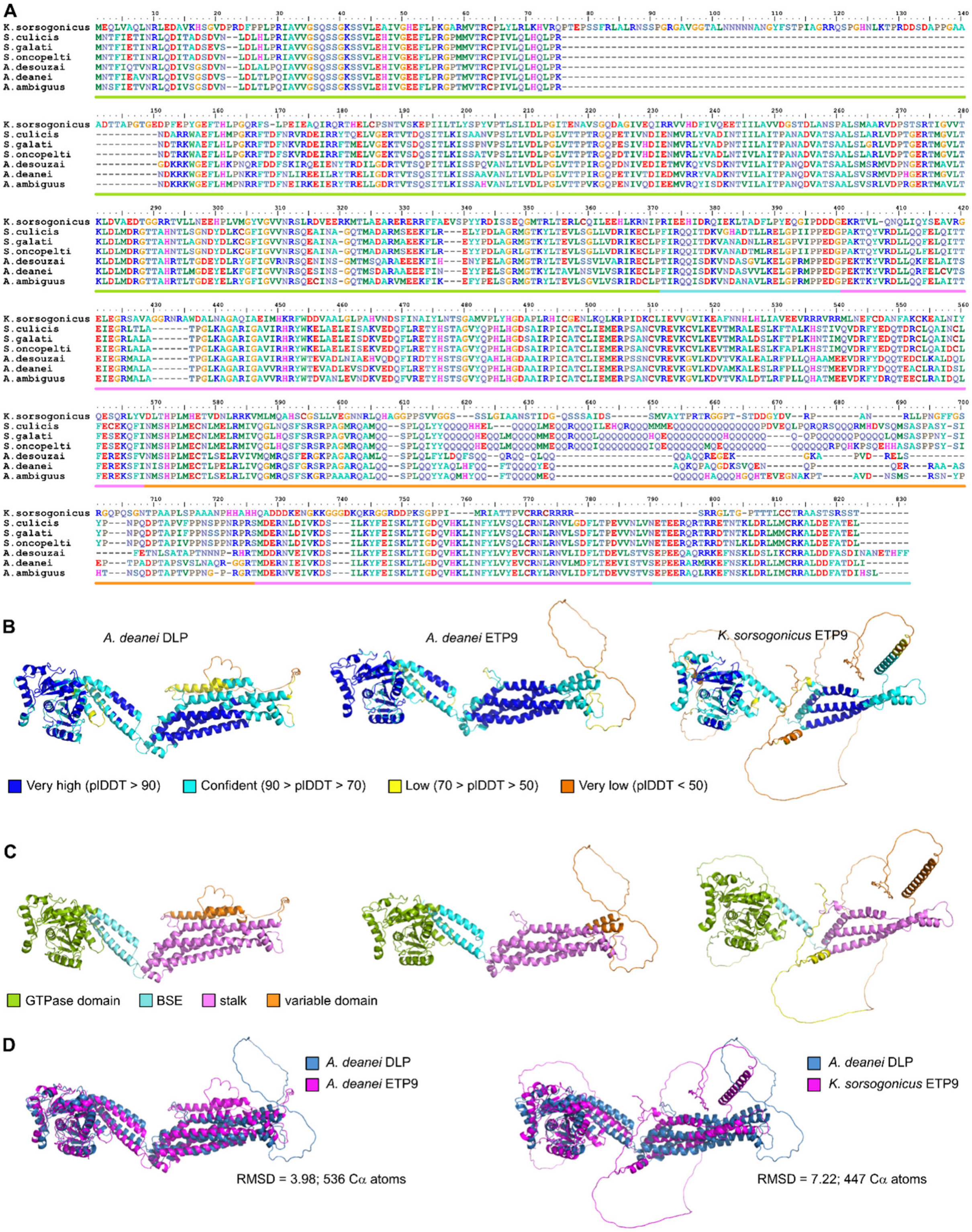

Figure S8: Conservation of ETP9 throughout the Strigomonadinae. A: ClustalO alignment of ETP9 amino acid sequences identified in the draft genome sequences available for members of the Strigomonadinae. DLP domain organization is indicated by a color code underneath the alignment (compare to panel C). Note the long insertion in the GTPase domain and low sequence conservation at the C-terminal stalk and BSE domains of *K. sorsogonicus* ETP9. Sequence identities: *K. sorsogonicus*, JAQOSZ010000001.1: 361472-363784; *S. culicis*, AUXH01000005.1: 71979-69808; *S. oncopelti*, AUXK01000002.1: 2417-249; *S. galati*, AUXN01000218.1: 12762-14927; *A. ambiguus*, JADNSB010000081.1: 64845-66935; *A. desouzai*, AUXL01000503.1: 3948-1900. **B:** Protein structures predicted by AlphaFold3 for the regular trypanosomatid DLP from *A. deanei* (*Ad*DLP; GenBank

accession CAD2218610.1), ETP9 (*Ad*ETP9; CAD2212698.1), and the ETP9 ortholog from *K. sorsogonicous* (*Ks*ETP9). Confidence scores of the prediction are mapped onto the protein structures. Note that for *Ad*DLP and *Ad*ETP9 only the variable domain is largely unstructured with low plDDT scores, whereas for *Ks*ETP9, for the variable domain plus the entire C-terminal part downstream of the variable domain no reliable structure can be predicted. **C:** Domain organization of the same proteins shown in C into GTPase domain, bundle signaling element (BSE), stalk, and variable domain (for details see Maurya et al. 2025). **D:** Overlay of the predicted structures of *Ad*DLP and *Ad*ETP9 (left) shows conservation of the typical DLP structure in *Ad*ETP9 with a root mean square deviation (RMSD) of 3.98 Å over 536 Cα atoms. Overlay of the predicted structures of *Ad*DLP and *Ks*ETP9 (right) shows much lower structural conservation of *Ks*ETP9 with an RMSD of 7.22 Å over only 447 Cα atoms.

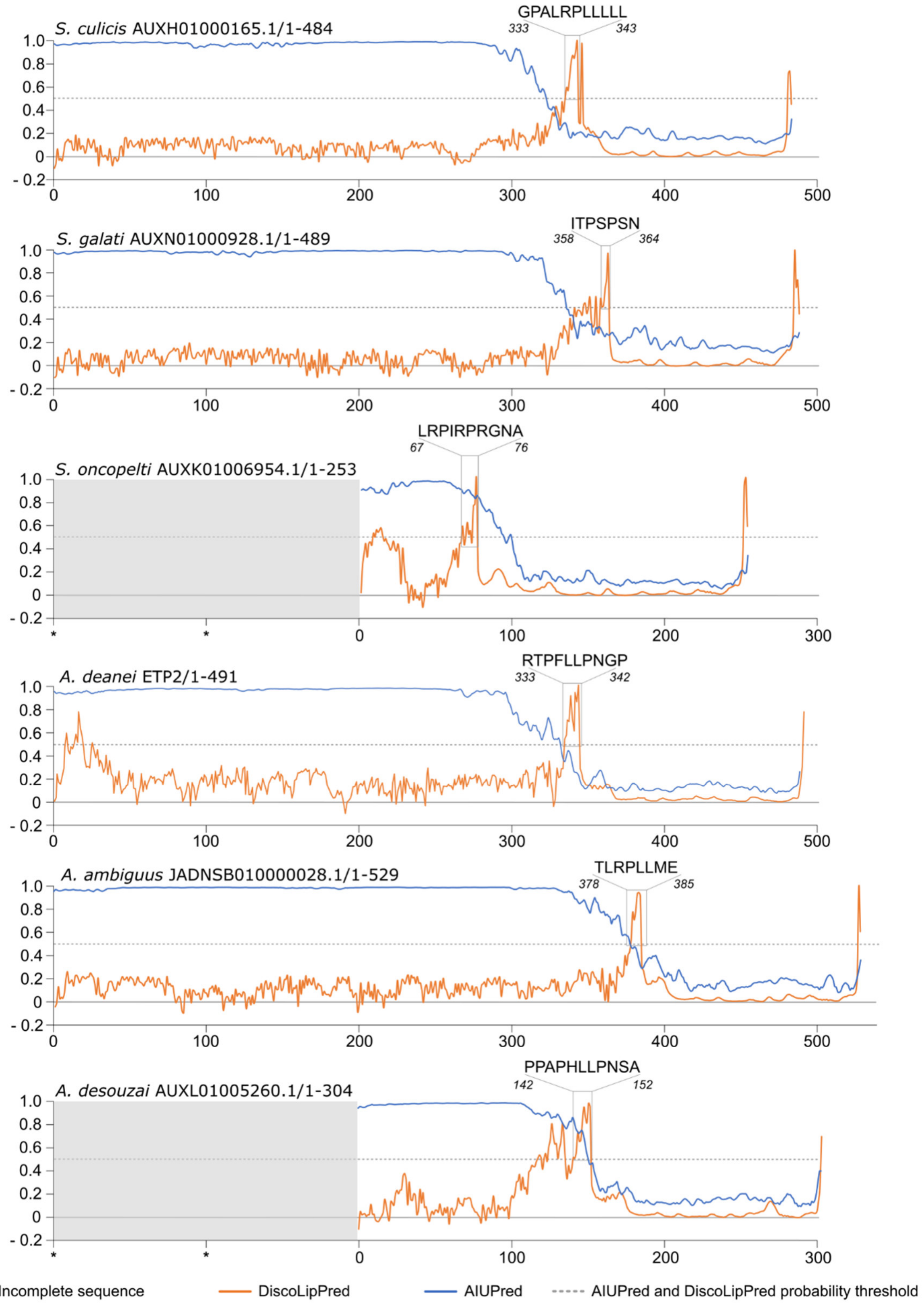

Figure S9: AIUPred and DisoLipPred analysis of ETP2 across Strigomonadinae. Graphs show AIUPred disorder prediction scores in blue and DisoLipPred lipid-binding probabilities in orange. Species name, contig accession, and protein length are indicated in the top left of each graph. The x-axis represents amino acid position, and the y-axis probability values, where 1 denotes the highest probability. For AIUPred, values below 0.5 (horizontal grey dotted line) indicate structured regions. For DisoLipPred, values above 0.5 suggest a high likelihood of disordered lipid-binding regions. The amino acid sequences corresponding to these predicted lipid-binding regions are highlighted in each graph. Grey-shaded plots denote species with an incomplete N-terminal sequence (see also **Fig. S7A**).

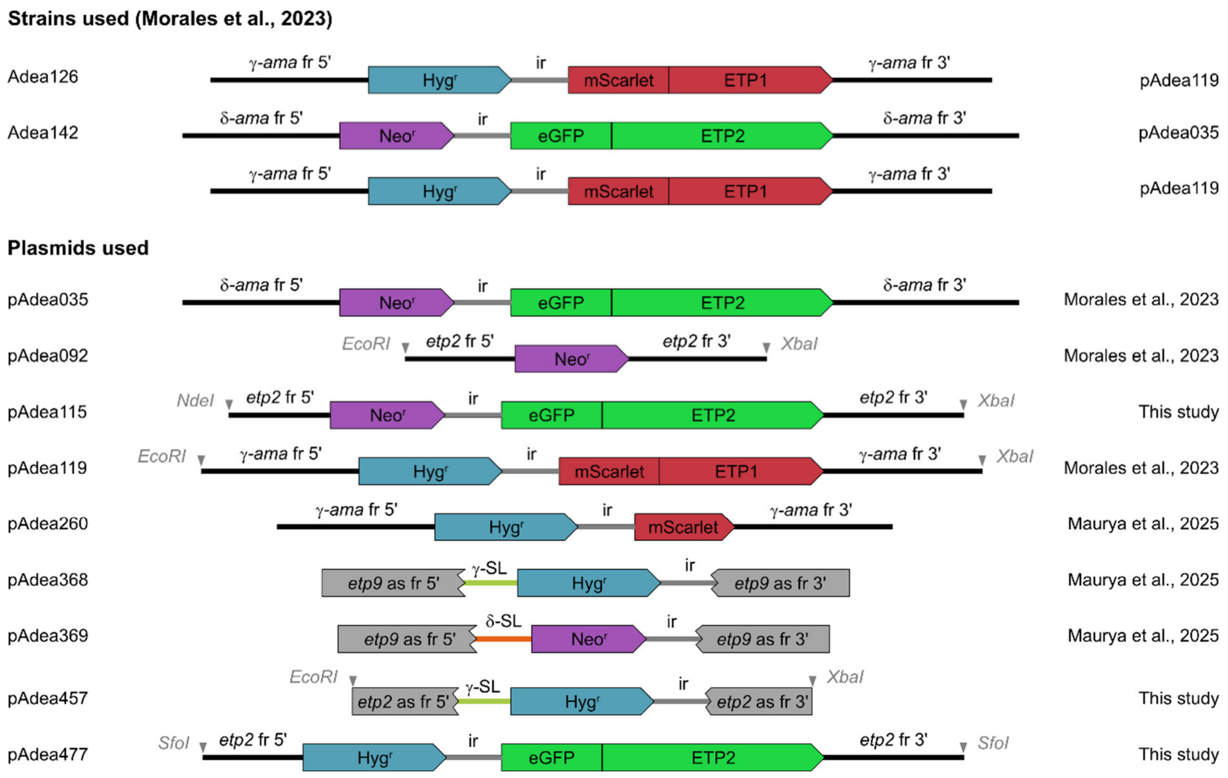

Figure S10: Schematic maps of all plasmids and *A. deanei* strains used in this study. Previously generated plasmids such as pAdea092 and pAdea119 were used in this study to generate new Adea strains or as templates to generate new plasmids in this study (pAdea260, pAdea368, and pAdea369). All other plasmids were generated in this study. Abbreviations: fr, flanking region (~0.7 - ~1.0 Kbp used for homologous recombination); ir, intergenic region between *gapdh I* and *gapdh II* genes; δ/γ-*ama*, δ- and γ-*amastin* gene (for nomenclature see Morales et al. 2016); *etp2* fr 5', upstream flanking region of *etp2* gene; *etp2* fr 3', downstream flanking region of *etp2* gene; *etp2/9* as fr 5', first half of the respective *etp* ORF used as 5' flaking region; *etp2/9* as fr 3', second half of the ORF used as 3' flaking region; δ/γ-SL, 5' flanking region of the δ/γ-*amastin* genes, respectively including the spliced leader acceptor sequence; Neo^{r}/Hyg^{r}, neomycin and hygromycin resistance markers encoded by *neo^{r}*/*hyg^{r}* genes, respectively. Arrowheads show approximate cutting sites of restriction enzymes on the plasmid used for linearization before transfection.

Table S1: All primers used in this study. Primers for the generation of plasmids, plasmid sequencing, verification of genomic insertions, and generation of Southern blot probes are described below.

Primer No.	Primer sequence (5'-3') Used for amplification of DNA fragment	Fragment amplified	Final plasmid
1015	GGTCTCCCTAATAATATATATTTATCTCGTTCGGTGTT	*etp2* fr 3'-pUMA1467-*etp2* fr 5'	pAdea115
1016	GGTCTCCTCATTTTGGTGTGTATGATTGTATTTTTC		
1022	GGTCTCCATGATTGAACAAGATGGATTGC	neo^r-gapdh ir-*egfp*-*etp2*	
1023	GGTCTCCTTAGTTCGCCGAAAGCAC		
3237	TCATCCTCGTCGTAGTCCATCAGGTGAGCTCGAATTCACT	pUMA1467	pAdea457
3244	TTGTGCTTTCGGCGAACTAAGCAGGTCTAGATATCGGATCC		
3238	AGTGAATTCGAGCTCACCTGATGGACTACGACGAGGATGAATACTTTGA	First half of *etp2* gene as fr 5'	
3239	CTAGGTGAGTTTTTACTTTTCAGTGCCTCTGGTGGGGT		
3242	CAAAAAACACAGTTATCCAACAGGTGCATCGTACGCCGG	Second half of *etp2* gene as fr 3'	
3243	GATCCGATATCTAGACCTGCTTAGTTCGCCGAAAGCACAAAATAGGGT		
3240	CACCCCCACCAGAGGCACTGAAAAGTAAAAACTCACCTAGTTTGC	γ-*ama* SL-hyg^r-gapdh ir	
3241	ACCGGCGTACGATGCACCTGTTGGATAACTGTGTTTTTTGATGAAA		
3320	ATACAATCATACACACCAAAATGAAAAAGCCTGAACTCACC	hyg^r	pAdea477
3321	TCAAACTCTCACTAGCACTTTTATTTCTTTGCCCTCGGAC		
3322	GTCCGAGGGCAAAGAAATAAAAGTGCTAGTGAGAGTTTGACT	pAdea115 excluding neo^r	
3323	GTGAGTTCAGGCTTTTTCATTTTGGTGTGTATGATTGTATTTTTCTATATA TATAA		
**Primer No.**	**Primer sequence (5'-3') Used for plasmid sequencing**	**Binding position**	**-**
55	ATCTTAGCCAGACGAGCG	hyg^r	-
56	CACTATCGGCGAGTACTTCTACA		-
117	TGCCGTCTCCTTCGTGAGCAAGGGCGAGGAG	*egfp*	-
118	TGCCGTCTCCTCTTACTTGTACAGCTCGTCCA		-
131	AAGTGCTAGTGAGAGTTTGACT	gapdh ir	-
132	TTGGATAACTGTGTTTTTTGATG		-
310	CGAAACATCGCATCGAGCG	neo^r	-
311	ATCGACAAGACCGGCTTCC		-
326	ACACGGCGACGGTGCCACCCCCACC	*etp2*	-
761	TGTAAAACGACGGCCAGT	pUMA1467	-
762	CAGGAAACAGCTATGACCAT		-
1066	GCGGTCCACCGATAACGTAAAGT	*etp2*	-
1440	CGCCGAGGTGAAGTTCGAGGGC	*egfp*	-
2873	CAGATTGTACTGAGAGTGCA	pUMA1467	-
**Primer No.**	**Primer sequence (5'-3') Used for PCR verification of genomic insertion**	**Locus/Gene**	**-**
80	CTTTCTGCCATCTGCCTCAT	γ-*amastin* (Bind in genome, outside of insertion cassette)	-
81	CATCCTTACGATCTTCTTATTTTTTGG		-
			-
			-
			-
55	ATCTTAGCCAGACGAGCG	hyg^r	-
1942	GCCCATTGTCTTCTTCTGCATT	*mscarlet*	-
1058	CTACACGTATTCCGTAGAGAG	*etp2*	-
1059	TAGAAGGAGAACACATTCCG		-
**Primer No.**	**Primer sequence (5'-3') Used for amplification of *etp2* gene**	**Gene**	**-**
3238	AGTGAATTCGAGCTCACCTGATGGACTACGACGAGGATGAATACTTTGA	*etp2*	-
3243	GATCCGATATCTAGACCTGCTTAGTTCGCCGAAAGCACAAAATAGGGT		-
**Primer No.**	**Primer sequence (5'-3') Used for Southern blot probe generation by nested PCR**	**Gene**	**-**
55	ATCTTAGCCAGACGAGCG	hyg^r	-
56	CACTATCGGCGAGTACTTCTACA		-
1302	GGTCTCAATGAAAAAGCCTGAACTCACC		-
1303	GGTCTCATTATTTCTTTGCCCTCGGACG		-

Table S2: Draft genome assemblies analyzed for the presence of orthologs of *etp2*, *etp7*, and *etp9*.

Species	Strain	GenBank Accession
Angomonas ambiguus	PNG-M02	GCA_019695575.1
Angomonas desouzai	TCC079E	GCA_000482185.1
Strigomonas culicis	TCC012E	GCA_000482145.1
Strigomonas galati	TCC219	GCA_000482125.1
Strigomonas oncopelti	TCC290E	GCA_000482165.1
Kentomonas sorsogonicus	MF-08	GCA_030347455.1
Wallacemonas collosoma	ATCC 30261	GCA_030849615.1
Wallacemonas rigidus	Sld	GCA_030849045.1
Wallacemonas sp.	MBr04	GCA_030849635.1
Wallacemonas sp.	TrypX	GCA_030849625.1
Wallacemonas sp.	Wsd	GCA_030849645.1
Wallacemonas sp.	195SL	GCA_030849605.1
Sergeia podlipaevii	CER4	GCA_030849805.1

Supplementary movies

Movie S1:

Cell co-expressing eGFP-ETP2 ($\Delta\delta\text{-}ama^{egfp\text{-}etp2}$) and mScarlet-ETP1 ($\Delta\gamma\text{-}ama^{mS\text{-}etp1}$) that is at an early stage of endosymbiont division showing a ring-shaped fluorescence signal around the ESDS when scrolling through confocal Z-stacks. Shown are individual channels for Hoechst 33342, mScarlet, and eGFP fluorescence, as well as a merge of all channels. Scale bar: 2 µm.

Movie S2:

Cell co-expressing eGFP-ETP2 and mScarlet-ETP1 that is at a later stage of endosymbiont division showing an x-like fluorescence signal at the ESDS when scrolling through confocal Z-stacks. Shown are individual channels for Hoechst 33342, mScarlet, and eGFP fluorescence, as well as a merge of all channels. Scale bar: 2 µm.
